# Design and evaluation of Actichip, a thematic microarray for the study of the actin cytoskeleton

**DOI:** 10.1186/1471-2164-8-294

**Published:** 2007-08-29

**Authors:** Jean Muller, André Mehlen, Guillaume Vetter, Mikalai Yatskou, Arnaud Muller, Frédéric Chalmel, Olivier Poch, Evelyne Friederich, Laurent Vallar

**Affiliations:** 1 Laboratoire de Biologie Moléculaire, d'Analyse Génique et de Modélisation, Centre de Recherche Public-Santé, 84 rue Val Fleuri, L-1526 Luxembourg, Luxembourg; 2 Laboratoire de Bioinformatique et Génomique Intégratives, Institut de Génétique et de Biologie Moléculaire et Cellulaire; Inserm, U596; CNRS, UMR7104, F-67400 Illkirch, Université Louis Pasteur, F-67000 Strasbourg, France; 3 Cytoskeleton and cell plasticity laboratory, Life Sciences RU, University of Luxembourg, 162a Avenue de la faïencerie, L-1511 Luxembourg, Luxembourg; 4 Computational Biology Unit, European Molecular Biology Laboratory, Meyerhofstrasse 1, D-69117 Heidelberg, Germany; 5 GERHM-Inserm U625, Université Rennes I, Campus de Beaulieu, Bt 13, Avenue du Général Leclerc, F-35042 Rennes cedex, France

## Abstract

**Background:**

The actin cytoskeleton plays a crucial role in supporting and regulating numerous cellular processes. Mutations or alterations in the expression levels affecting the actin cytoskeleton system or related regulatory mechanisms are often associated with complex diseases such as cancer. Understanding how qualitative or quantitative changes in expression of the set of actin cytoskeleton genes are integrated to control actin dynamics and organisation is currently a challenge and should provide insights in identifying potential targets for drug discovery. Here we report the development of a dedicated microarray, the Actichip, containing 60-mer oligonucleotide probes for 327 genes selected for transcriptome analysis of the human actin cytoskeleton.

**Results:**

Genomic data and sequence analysis features were retrieved from GenBank and stored in an integrative database called Actinome. From these data, probes were designed using a home-made program (CADO4MI) allowing sequence refinement and improved probe specificity by combining the complementary information recovered from the UniGene and RefSeq databases. Actichip performance was analysed by hybridisation with RNAs extracted from epithelial MCF-7 cells and human skeletal muscle. Using thoroughly standardised procedures, we obtained microarray images with excellent quality resulting in high data reproducibility. Actichip displayed a large dynamic range extending over three logs with a limit of sensitivity between one and ten copies of transcript per cell. The array allowed accurate detection of small changes in gene expression and reliable classification of samples based on the expression profiles of tissue-specific genes. When compared to two other oligonucleotide microarray platforms, Actichip showed similar sensitivity and concordant expression ratios. Moreover, Actichip was able to discriminate the highly similar actin isoforms whereas the two other platforms did not.

**Conclusion:**

Our data demonstrate that Actichip is a powerful alternative to commercial high density microarrays for cytoskeleton gene profiling in normal or pathological samples. Actichip is available upon request.

## Background

The actin cytoskeleton is a highly dynamic network of protein polymers extending throughout the cytoplasm. It not only provides structural support for the cell, but also plays a central role in key cell processes including cellular morphogenesis, migration, division and cell communication. The actin cytoskeleton generates forces required for membrane extension and remodelling, motor protein-dependent cell contraction or membrane trafficking [1]. Recently, a nuclear function was identified for actin in the organisation of chromatin and gene expression [2,3]. In cells, the assembly and disassembly of actin filaments and their organisation into higher-order networks is regulated by actin-associated proteins which, in turn, are controlled by specific signalling pathways [1,4]. The formation of membrane-cytoskeleton specialisations not only depends on the spatio-temporal controlled recruitment of actin-binding proteins to cellular subdomains, but also on the repertoire of specific sets of cytoskeleton and regulatory proteins that cells express at a given state. In line, timely and spatially regulated expression of cytoskeletal genes is observed during embryonic development or terminal differentiation of cells in adults.

The central role of the actin cytoskeleton in many essential cellular processes makes the system susceptible to mutations and alterations of gene expression level that may cause a wide range of diseases, including muscular dystrophies, amyloidosis, haematological disorders and cancers [5,6]. Many of these diseases arise from aberrant cell morphogenesis, motility or communication caused by deregulation of actin dynamics or organisation. For example, deregulated cell motility is a typical hallmark of tumour invasion and metastasis characterising cancer malignancy. Recent studies demonstrated that tumour cell progression correlates with alterations of the expression profile of actin cytoskeleton genes and genes of upstream regulatory pathways [6-8]. Similarly, altered expression of genes encoding cytoskeletal proteins of the contractile system of muscle cells is observed in cardio-vascular disorders like heart failure [9]. Therefore, cytoskeleton proteins are potential markers for cell differentiation or disease, and might constitute promising novel targets for therapeutic treatments [10].

The basic set of structural and signalling protein components of the actin cytoskeleton is now identified and information on their biochemical or biological activities is available. However, gaps and controversies remain on how qualitative or quantitative changes in expression of these proteins are integrated to control actin dynamics and organisation in space and time. Elucidating the intricate interplay between the cytoskeletal components that cells use to build-up various cellular structures is hampered by the complexity of the actin cytoskeleton system. In this context, gene expression profiling using microarrays has the potential to yield a global overview on the set of actin cytoskeleton genes expressed by a cell at a given physiological or pathological state. The technique allows global and parallel investigations of cellular activity, and was used successfully to characterise the molecular basis of a variety of complex experimental models and diseases. Results obtained in previous profiling studies with high-density microarrays underline the potential of this approach for detecting changes in the repertoire of expression of the cytoskeleton genes [7,8].

Using an optimised experimental approach, we developed Actichip, a custom oligonucleotide microarray designed to study the expression of actin cytoskeleton genes in various cell systems. Actichip represents 327 human genes, most of them encoding proteins that bind directly to actin and control actin dynamics or organisation, while the others are involved in signalling, cell-cell or cell-matrix adhesion. In parallel, we developed Actinome, a knowledge database that integrates information on the target genes, including genomic data and sequence analysis features retrieved from GenBank, and biological function, when available. We determined the performance characteristics of Actichip and compared them with those of two other academic or commercial oligonucleotide arrays. Our data indicate that Actichip exhibits solid performance that makes it a valid platform for studying the human transcriptome of the actin cytoskeleton.

## Results

### Actinome database

To facilitate the setting up of Actichip, an integrative database called Actinome was implemented cataloguing genomic data and sequence analysis features of the human genes related to the actin cytoskeleton. We also considered some key marker genes including adhesion receptors, metalloproteases or extracellular matrix proteins that are involved in actin-based proccesses like morphogenesis or cell migration. Gene selection was performed using the GenBank database (release 134,[11]), and was based on a combination of biological knowledge, literature data, Gene Ontology (GO) terms [12] and keywords in the NCBI database. Searches were restricted to genes encoding proteins of the major functional groups regulating the dynamics and organisation of the actin cytoskeleton (Table 1). Actinome was built following a robust protocole as described in the "Method" section. To date, the database compiles a set of 327 non redundant entries and related data such as mRNA and protein identifiers, gene names and descriptions, cytobands and gene ontology annotations. Actinome is freely available [13].

**Table 1:** Functional groups of genes represented on the Actichip microarray

Gene function category		Number of genes represented on Actichip	Number of probes
**Actin cytoskeleton**	Actins	7	8
	Actin nucleators	9	14
	Actin bundling (cross-linking, severing)	52	56
	Actin sequestrating	7	7
	Barbed end actin capping	6	6
	Pointed end capping	7	7
	Filamentous actin stabilisation	5	5
	Actin depolymerisation	11	11
	Actin-dependent motors	42	44
	Actin-related, actin-like proteins	16	16
	Actin-binding proteins	14	14
	Cytoskeletal proteins	21	21
	Plasma membrane cortical cytoskeleton linkers/scaffold, signalling	52	60

**Actin-based processes**	Tissue-specific	4	5
	Cell adhesion	40	49
	Cancer	12	12
	Signalling	10	10
	DNA, transcription and translation factors	12	14

**Total**		**327**	**359**

A set of 359 long oligonucleotides (60-mer) were designed to represent 249 genes encoding proteins implicated in the actin cytoskeleton machinery or 78 genes involved in actin-based processes. Most of the genes (301) are represented by a unique probe, 22 genes by two probes, 3 genes by 3 probes and one gene by 5 probes.

### Oligonucleotide probe design

We decided to use long oligonucleotide probes to build Actichip because of the numerous advantages they offer when compared to PCR amplicons. Being fully custom-designed, they have more uniform hybridisation characteristics, they yield less non-specific hybridisation and misidentification of gene transcripts, while exhibiting similar sensitivity [14].

Genomic databases are still prone to modifications. While reorganisations and changes in transcript sequences or identifiers may account for erroneous results in microarray studies, using sequence-verified probes was shown to improve microarray measurement accuracy and consistency [15,16]. Therefore, we decided mining transcript information for probe design from several databases. Although many programs to design oligonucleotide probes were publicly available at the time of our study [17-23], none of these programs allowed such an application. Therefore, we implemented a new, freely available program named CADO4MI (Computer-Assisted Design of Oligonucleotides for Microarrays) [24]. As most of the existing programs, CADO4MI uses variations of the same algorithm and common criteria to design specific oligonucleotides with optimised hybridisation features through a multistep procedure (see "Methods"). Contrary to these programs however, CADO4MI has the potential to compute probe sets for the same query genes using simultaneously two or more databases in order to select optimal sequences. Visualisation, comparison and integration of the different probe sets are greatly facilitated by a powerful graphical user interface. The program also incorporates several other interesting features such as an automatic search for missing sequences in the reference databases and the possibility to compute and display melting temperature (Tm) or GC content curves for individual gene query or, alternatively, for the entire set of sequences. These curves are helpful in selecting the appropriate parameters for the design of probes. CADO4MI was used successfully in a recent study to select automatically a set of PCR primers designed for the resequencing of Interrupted CoDing Sequences (ICDS) [25].

We designed 60-mer oligonucleotides using the Reference Sequence database (RefSeq) [26] and the UniGene database [27] because of their complementary features. While the former gives access to non-redundant and well-annotated sequences including pseudogenes and splice variants, but is not yet exhaustive, the latter compiles comprehensive gene-oriented clusters of sequences but with more redundancy and incomplete or erroneous annotations. The Actichip probe set was designed to target each of the 327 genes defined in the Actinome database with a single oligonucleotide without discriminating splice variants. This was achieved for 301 entries while the probes designed for 26 genes were shown to target more than one sequence in at least one reference database. We therefore selected either 2 oligonucleotides for 22 genes, 3 for 3 genes or 5 for one gene resulting in a total set of 359 oligonucleotides (Table 1). In addition, we used part of a set of viral and bacterial probes described as having no similarity with human transcripts [28] and sequences of human genes reported as being housekeeping genes [29] to generate 41 negative and 32 positive controls, respectively.

### Evaluation of Actichip performance

#### Experimental design

To evaluate the experimental performance of Actichip microarrays, a series of repeated hybridisation experiments, including dye swaps, were carried out with optimised target labeling, hybridisation, scanning and data analysis protocols (see "Methods"). All the procedures were standardised to limit the impact of experimental bias or biological variations on data. The same series of high quality RNA samples purified from human breast adenocarcinoma MCF-7 cells and from human skeletal muscle was used in our experiments. Epithelial cells and skeletal muscle tissue were chosen because they express well-characterised sets of cytoskeleton genes, and were anticipated to give well-contrasted differential expression data when analysed with Actichip.

#### Actichip image quality and data reproducibility

**Figure 1:**
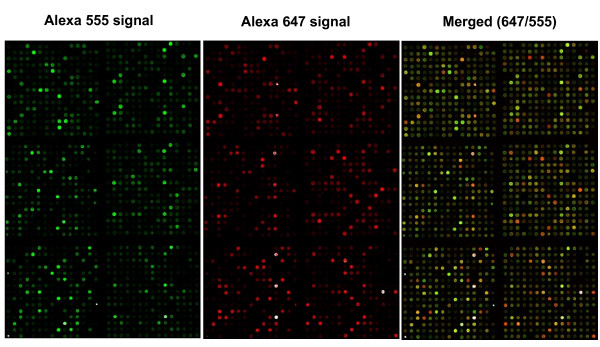
**The Actichip microarray **is composed of 6 subgrids each containing 15 × 15 spots, resulting in a total of 1350 spots. Images were obtained following hybridisation of fluorescently-labeled samples derived from MCF-7 cells (green channel) or human skeletal muscle (red channel), and are representative of ten replicates.

Microarray images exhibited good and reproducible quality parameters in both channels (Figure 1). Background values were low and signals showed maximum dynamic range, resulting in signal-to-background and signal-to-noise ratios higher than 20 and 50 in each channel, respectively. Actichip microarray images were quantified using the Genepix Pro 6.0 software. Negative and irrelevant spots were removed from the dataset as described in the "Methods" section, yielding approximately 80 % of positive features of which 55–60 % were found to be relevant.

**Figure 2:**
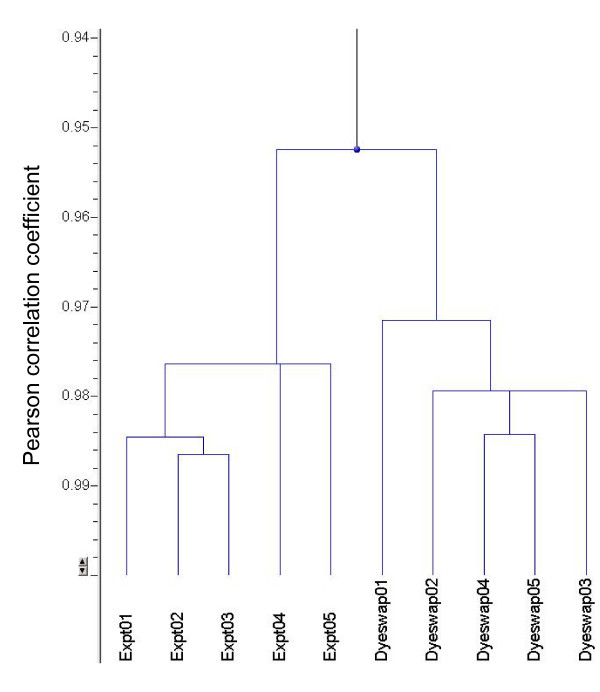
**Analysis of the reproducibility of Actichip data using a hierarchical array clustering**. The array data from ten replicates were clustered in the Acuity program using a hierarchical procedure based on the average linkage method and Pearson correlation coefficient. The experiments are grouped on the resulting dendogram according to their relative degree of similarity. Experiments and dye swaps 1 to 3 and experiments and dye swaps 4 and 5 were performed at two different time periods.

To assess the reproducibility of Actichip data, we analysed triplicate spots on the Actichip array (intra-array reproducibility) and repeated hybridisations (technical inter-array reproducibility). Focusing on the intrinsic performance of Actichip, we did not investigate the impact of the hybridisation of different samples (biological inter-array reproducibility). We calculated the standard deviation (STD) and coefficient of variation (CV) between the normalised Log_2 _ratios values excluding the irrelevant signals. As shown in Table 2, the microarrays gave reproducible results with good intra- and inter-assay STD and CV. Analysis of variance (ANOVA test) showed that the series of normalised signal ratios was highly similar (p < 0.05). The variability in data was also examined in the Acuity 4.0 program using the hierarchical clustering of array experiments with the average linkage and the Pearson correlation coefficient as similarity metric. Two main clusters were identified for correlation coefficients > 0.95 in the dendogram resulting from this analysis, each corresponding to either normal or dye-swap experiments (Figure 2). The correlation coefficients calculated from microarray hybridisations ranged from 0.95 to 0.99 indicating that the assays were highly comparable. Data from assays performed at different time periods were undistinguishable. Although limited, the greatest source of variability in our dataset was the dye exchange, indicating that a slightly uneven incorporation of the dyes in samples occurred during our experiments. This dye bias was compensated through a ratio-based, global normalisation of the data.

**Table 2:** Reproducibility of the Actichip array data

Measurements	Median STD	Median CV
Within array replicates^a^	0.065	4.275 %
Inter-array repeats^b^	0.103	6.228 %

(a) Intra-array reproducibility was estimated from two representative array experiments performed simultaneously (one standard and one dye-swap). Standard deviation (STD) and coefficient of variation (CV) of Log_2 _signal ratios were calculated for each measurable triplicate spots, and the mean values of all repeated measurements were computed for each array. The median of mean STDs and CVs obtained from the two arrays are indicated. (b) Inter-array reproducibility as determined from 10 arrays performed at two distinct time periods (5 standard and 5 dye-swap). Median STD and CV were calculated from the mean Log_2 _signal ratios obtained for each measurable gene over the series of arrays.

#### Signal linearity and detection limit

To investigate the dynamic range of Actichip and to determine the span of this dynamic range, we used seven *Arabidopsis thaliana *polyadenylated RNA species referred to as spike RNAs that were *in vitro *synthesised from plasmids provided by the Institute for Genomic Research [30]. The spike RNAs were calibrated and were combined to construct seven complex mixes, each mix containing six of the seven spike RNAs in staggered concentration ranging from 10^-1 ^to 10^4 ^copies per cell (cpc). An eighth sample was prepared, called the reference sample, consisting of the mix of all spike RNAs at a concentration of 10^2 ^cpc [see Additional file 1]. Thereby, the comparison of any of the seven RNA samples to the reference sample should theoretically yield signal ratios ranging from 10^-3^-fold to 10^2^-fold.

**Figure 3:**
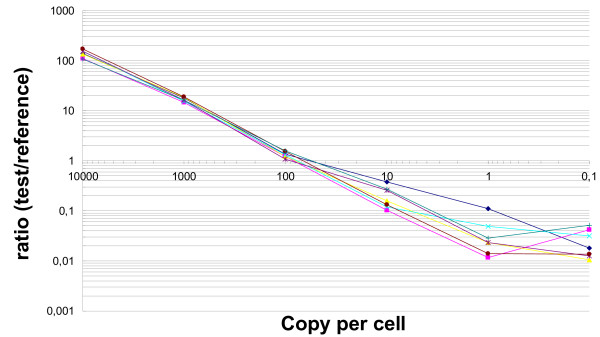
**Dose-response curve for the Actichip array**. Each individual *A. thaliana *spike mix was compared directly to the reference mix, resulting in seven hybridisations. For each hybridisation series, the raw signals were pre-processed according to the procedure detailed in the Methods section. Array data were normalised using a median Log_2 _ratio-centring approach after subtraction of the local median background intensity from the median foreground intensity. The normalised Log_2 _ratios were averaged over 3 replicates, and the final ratio was computed as the exponential base 2 of this average. The abscissa indicates the spike RNA abundance expressed as copy per cell (cpc) in the seven spike mixes. The ordinate shows the resulting normalised intensity ratios relative to the reference mix (all spikes at a concentration of 100 cpc). The data are mean values from three independent experiments.

The graph shown in Figure 3 is a summary of a complete hybridisation series where each curve represents the signal ratios associated with one of the seven spike RNAs. Actichip arrays displayed a near perfect dynamic range over three logs (10–10^4 ^cpc) and the experimental Log_2 _ratios match well the expected theoretical values. A wider spread of the curves was observed for some spike RNAs indicating that their sequences might favour hybridisation signal accuracy. Actichip arrays accurately yielded ratios for spike RNAs at the highest concentration (10^4 ^cpc, 10^2^-fold ratio) with no saturation effect. The lower limit of linearity of the dynamic range was around 10 cpc, and the signals reached a bottom plateau with values close to background noise, marking the limit of sensitivity between 1 and 10 cpc.

#### Actichip reliability

To validate the reliability of Actichip data, we analysed our dataset using the Significance Analysis of Microarrays algorithm (SAM; [31]). SAM analysis resulted in a list of 106 and 176 genes found significantly expressed in skeletal muscle and MCF-7 cells, respectively (Δ = 1.85; FDR = 0 %). This list was highly enriched in marker genes characteristic for either epithelial or skeletal muscle cells (Table 3), in good agreement with the expression patterns expected from an *a priori *reasoning based on biological knowledge. Importantly, we obtained similar results through SAM analysis using three randomly chosen experiments over the entire series of assays (data not shown) revealing that a limited number of repeats would be sufficient to obtain reliable data with Actichip.

**Table 3:** List of the top 15 most significantly expressed genes in MCF-7 cells or in skeletal muscle as determined using the Actichip microarray

	Gene name	Gene Accession number	Score (d)	Log_2 _expression ratio
Genes mainly expressed in skeletal muscle	Nebulette (Actin-binding Z-disk protein)	NM_006393	42.93	3.81
	Troponin I, fast skeletal muscle (Troponin I, fast-twitch isoform)	NM_003282	34.39	2.60
	Huntingtin interacting protein 1 (HIP-I)	NM_005338	33.55	2.59
	Myosin light chain 2a	NM_021223	32.80	3.26
	Troponin C, skeletal muscle	NM_003279	32.21	2.45
	Myosin light chain 2	NM_013292	29.25	3.06
	Myosin heavy chain, skeletal muscle, perinatal	NM_002472	28.14	2.80
	Troponin T, fast skeletal muscle isoforms	NM_006757	26.04	2.71
	Myosin heavy chain, cardiac muscle beta isoform	NM_000257	24.94	3.12
	Actin, alpha skeletal muscle (Alpha-actin 1)	NM_001100	24.21	3.50
	Troponin C, slow	NM_003280	21.31	2.54
	Nebulin	NM_004543	20.17	2.78
	Smoothelin	NM_134269	19.50	1.32
	Tropomyosin 1 alpha chain (Alpha-tropomyosin)	NM_000366	18.92	1.99
	Myotilin	NM_006790	18.07	1.56
Genes mainly expressed in MCF-7 cells	Actin, cytoplasmic 2 (Gamma-actin)	NM_001614	-30.24	-2.58
	Elongation factor 1-beta (EF-1-beta)	NM_001959	-22.14	-1.26
	Actin binding protein anillin,	NM_018685	-20.12	-1.13
	Keratin, type I cytoskeletal 18	NM_000224	-18.78	-2.83
	Ankyrin 3 (ANK-3) (Ankyrin G)	NM_020987	-16.43	-2.17
	Gamma-parvin	NM_022141	-16.30	-1.03
	Spectrin beta chain, brain 2 (Spectrin, non-erythroid beta chain 2) (Beta-III spectrin)	NM_006946	-16.28	-0.84
	Coronin 1B (Coronin 2)	NM_020441	-15.97	-0.84
	Beta-centractin (Actin-related protein 1B) (ARP1B)	NM_005735	-15.79	-1.40
	NB thymosin beta	NM_021992	-14.92	-1.42
	Coronin 2A (WD-repeat protein 2) (IR10)	NM_052820	-14.39	-1.24
	ARP2/3 complex 16 kDa subunit (P16-ARC) (Actin-related protein 2/3 complex subunit 5)	NM_005717	-13.74	-1.06
	Tight junction protein ZO-1 (Zonula occludens 1 protein) (Zona occludens 1 protein) (Tight junction protein 1)	NM_003257	-13.70	-1.28
	Transcription factor 7 (T-cell-specific transcription factor 1) (TCF- 1) (T-cell factor 1)	NM_003202	-13.60	-0.67
	Catenin delta-1 (p120 catenin) (p120(ctn)) (Cadherin-associated Src substrate) (CAS) (p120(cas))	NM_001331	-13.60	-0.77

The gene list was established from our dataset through SAM analysis [64]. In SAM, the significant genes are represented on a plot by points displaced by a distance denoted as the score (d) from a line on which genes identified simply by chance are aligned. Log_2 _expression ratios were determined by averaging the Log_2 _ratios calculated from fluorescence intensities of 10 repeated hybridisations.

#### Actichip specificity

The major difficulty in designing oligonucleotide probes for Actichip arised from the appreciable number of highly similar genes found in the actin cytoskeleton gene family [32]. This is exemplified by the actin gene family which is composed of six different isoform genes sharing not only high sequence identity at the protein level (> 95% Id.), but also at the nucleic acid level (> 85% Id.). For these genes, sequence identity ranges between 91 and 99% of the Coding Sequence (CDS), and between 57 and 83% of the total mRNA sequence hampering the design of specific probes.

**Figure 4:**
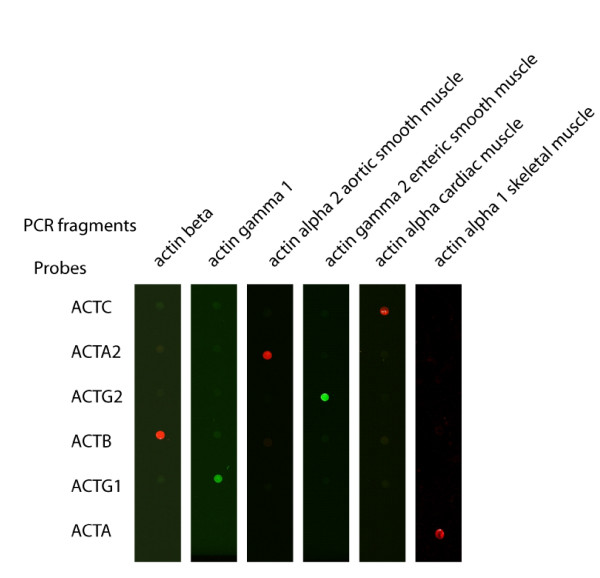
**Discrimination of the actin isoforms by Actichip**. PCR fragments corresponding to the six actin isoforms were generated from cDNA and were fluorescently labeled. The figure shows the images resulting from the specific hybridisations of the PCR fragments onto independent Actichip arrays. ACTC1: Actin, alpha, cardiac muscle 1; ACTA2: Actin, alpha 2, smooth muscle, aorta; ACTG2: Actin, gamma 2, smooth muscle, enteric; ACTB: Actin, beta; ACTG1: Actin, gamma 1; ACTA1 Actin, alpha 1, skeletal muscle.

To evaluate the specificity of the Actichip probes targeting the actin isoforms, PCR fragments corresponding to the target regions of the transcripts were obtained using as template cDNA generated from Hela cell poly(A)RNA. The purified PCR products were labeled with Alexa dyes through direct covalent linkage and hybridised to Actichip microarrays. As shown in Figure 4, each PCR fragment bound to the corresponding probe. No cross-hybridisation was observed neither within the set of actin probes nor with the other oligonucleotides included in the chip. These results demonstrated that the oligonucleotide probes were fully specific and indicated that the procedure used to design the probes in CADO4MI was robust.

### Cross platform comparison

To further evaluate the performance of Actichip, we compared the microarray with two other well-established oligonucleotide-based platforms (Table 4). We used 25-mer oligonucleotide commercial chips (Affymetrix, human genome U133A 2.0) and academic arrays prepared with a 70-mer oligonucleotide set (Operon, human whole genome version 2.0). PCR-amplicon arrays were not considered to avoid bias generated by this format of probe, as a result of either sequence errors or cross-hybridisations [33]. Hybridisation replicates were carried out under optimised and standardised protocols specific to each platform using the RNA sample sets analysed with Actichip. Microarray image analysis and data extraction were performed using dedicated methods and software (see "Methods").

**Table 4:** Microarray platform features

**Microarray characteristics**	**HG-U133A 2.0 (Affymetrix)**	**Human oligonucleotide set version 2.0 (Operon)**
Probe length	25-mer	70-mer
Number of probes/probe sets	> 22000	25392 including 3871 controls
	Genbank	
Main databases used for the design	dbEST	RefSeq
	RefSeq	UniGene (build Hs 147)
	UniGene (build Hs 133)	
Number of target transcripts	18400 including variants	21329 including :11530 designed from UniGene and RefSeq
Number of genes represented	14500	9799 designed from UniGene
Number of probes/target sequence	11	1

The table summarises the main characteristics of the two microarray platforms compared to the Actichip array. Data are from the description facts provided by the manufacturers.

**Figure 5:**
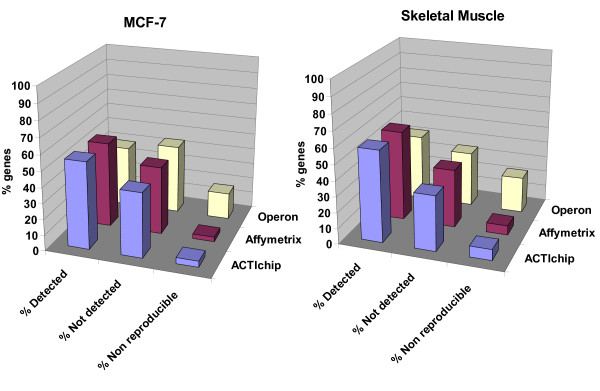
**Microarray platform comparison**. Series of expression profiling assays were performed using the HG-U133A 2.0 Affymetrix GeneChip (3 replicates per RNA sample), the human oligonucleotide Operon set (4 replicates) and the Actichip arrays (10 replicates). Experiments were carried out using the same lots of RNAs extracted from human carcinoma MCF-7 cell line and from human skeletal muscle. Data were analysed as stated in the "Methods" section resulting in two groups of genes : "detected" and "not detected". The genes found similarly expressed or not expressed in less than 2/3 of the replicated assays were deemed "non reproducible". The histograms show the distribution of the three groups of genes for each platform relative to the samples. Results are expressed as percentages relative to the total number of genes simultaneously represented on each array platform.

Results obtained with Actichip, Affymetrix and Operon arrays were markedly comparable (Figure 5). The percentage of probes found to be relevant in Genepix ranged from 53 to 55 % for MCF-7-derived RNAs whereas it varied from 57 to 60 % for the skeletal muscle sample. Compared to the Operon platform, the Actichip and Affymetrix arrays gave the best inter-assay reproducibility with only 5 % of discordant data between the series of experiments.

Cross-platform comparison was achieved considering only the genes represented simultaneously on each of the three arrays. These genes were identified by comparing the target gene accession numbers and/or the sequences used to design the probes as detailed in the "Methods" section. Among the 327 genes represented on Actichip, 304 were also targeted by the Affymetrix U133A 2.0 array and 294 by the Operon array, while 275 genes were represented simultaneously on the three platforms. We calculated the degree of concordance between the expression patterns as the ratio of the number of genes simultaneously found expressed or not expressed in our samples by two or three of the microarray platforms to the total number of genes commonly represented by these platforms. We found 49 % of concordant genes between Actichip and Affymetrix, 35 % between Actichip and Operon, 45 % between Affymetrix and Operon, and 24 % when considering all platforms. Results were comparable for the two RNA samples we analysed. The Pearson correlation coefficients (r) calculated using the median expression Log_2 _ratios of the set of concordant genes were 0.88 between Actichip and Affymetrix, 0.63 between Actichip and Operon, and 0.67 between Affymetrix and Operon. These data indicated that Actichip microarrays performed equally well as Affymetrix platform while Operon arrays were less reliable under our experimental conditions.

As a good indicator of platform specificity, we found that the expression patterns obtained with Actichip for the actin isoforms perfectly matched the profiles described in the literature [34]. Cytoplasmic actin isoforms (ACTB and ACTG1) were detected in both samples as anticipated from their ubiquitous expression (Table 5). Two of the four muscle actin isoforms (ATCA1 and ACTC1) were found to be expressed in skeletal muscle but not in MCF-7 cells while the others (ACTA2 and ACTG2) were not detected in any sample. These expression patterns were further confirmed by PCR using cDNAs obtained from our samples (data not shown). Conversely, the aortic smooth muscle (ACTA2) and gamma enteric smooth muscle (ACTG2) actin isoforms were incorrectly identified in the MCF-7 and skeletal muscle samples using the Affymetrix chips. With the Operon arrays, the alpha skeletal muscle (ACTA1) and aortic smooth muscle (ACTA2) actins were inaccurately detected in the MCF-7 or skeletal muscle RNAs, respectively. In addition, Operon arrays were unable to detect the gamma actin (ACTG1) in both samples.

**Table 5:** Microarray platforms differentially discriminate the various actin isoforms

Actin isoforms	Actichip		Affymetrix		Operon	
	
	MCF-7	Skeletal muscle	MCF-7	Skeletal muscle	MCF-7	Skeletal muscle
Actin, alpha 1, skeletal muscle (ACTA1)	-	+	-	+	+	+
Actin, alpha 2, smooth muscle, aorta (ACTA2)	-	-	+	+	-	+
Actin, beta (ACTB)	+	+	+	+	+	+
Actin, alpha, cardiac muscle 1 (ACTC1)	-	+	-	+	Ø	Ø
Actin, gamma 1 (ACTG1)	+	+	+	+	-	-
Actin, gamma 2, smooth muscle, enteric (ACTG2)	-	-	+	+	-	-

Hybridisation data were treated following the procedure specific for each platform (see "Methods") to identify genes reproducibly deemed expressed (+) or not expressed (-). The table summarises the expression data obtained for the different actin isoforms. Ø : Isoform not represented on the array.

## Discussion

Microarray analysis is a powerful methodology for high throughput gene expression study which contributes to the understanding of complex events or biological systems [35]. In the present paper we describe the design and benchmarking of a custom-made oligonucleotide microarray named Actichip as a tool to study the actin cytoskeleton in normal or pathological situations.

We designed, produced and evaluated Actichip using optimised and standardised experimental procedures and a data evaluation pipeline we established according to the guidelines developed by the Microarray Gene Expression Data (MGED) Society [36]. Actichip hybridisation signals obtained with our optimised experimental settings were of high quality (Figure 1) leading to accurate and highly reproducible quantification of gene expression levels (Table 2, Figure 2). Importantly, our data indicated that two or three replicates would be sufficient for reliable measurements when applying the standardised procedures we established. Consistent with recent studies [37-40], our results show that a thorough standardisation of the array and experiment design, protocols and data analysis procedures, can greatly improve microarray data quality and comparability. This is crucial for the generation of meaningful universal gene expression index based on the exchange and integration of data between microarray platforms and laboratories.

The reliability and sensitivity of gene expression measurements are other important issues when using microarrays. In this study, we analysed two well-contrasted RNA samples, each characterised by a specific organisation of their actin cytoskeleton and by known marker genes. Many of these genes were found significantly expressed using Actichip (Table 3), underlining the reliability of this array as a transcriptome analysis platform and its value for the characterisation and classification of biological samples based on their transcriptome profiles. Our data further showed that Actichip not only detects reliably qualitative gene expression changes, but has also the potential to accurately measure the amplitude of these variations (Figure 3). In addition, we determined that Actichip has the potential to identify transcripts over a biologically meaningful range including high, intermediate and rare abundance classes of RNAs.

The fraction of probes on an array that yield a significant hybridisation signal can be used as a measure of platform sensitivity. We found a magnitude of detectable genes ranging from 53 to 60 % with both the Actichip, Affymetrix and Operon microarrays (Figure 5), indicating that the reactivity of the three platforms is similar. These results are in good agreement with data from similar studies [16,41], and suggest that a significant fraction of cytoskeletal genes were not or very lowly expressed in our samples, consistent with the concept that only part of the genome is usually expressed in a given differentiated cell line or tissue [42].

Comparison of the expression profiles obtained from the three platforms revealed a moderate concordance between the datasets, the best score (49 %) being observed between the Actichip and Affymetrix arrays. Nevertheless, we found good correlations between the relative expression data from the different arrays when considering the subset of concordant genes. The correlation in gene expression levels between the Actichip and Affymetrix arrays was particularly strong and was comparable to those reported in similar studies for best performing arrays [16,43-46]. Identifying the source of variability between the different microarray platforms was not straightforward since many factors could have influenced the expression data. Indeed, microarray platforms differ on numerous technological aspects including array format and fabrication, protocols and instrumentation, as well as computational and statistical tools. It has been shown that these differences could account, at least in part, for discrepancy in the data generated by different array technologies [33,47-50]. Although we carefully standardised our protocols, we could not avoid some differences in the procedures specific for each platform. Biases in our data may partly result from dissimilarities between the methods we used to generate and label the samples or from differences in sensitivity between the procedures we applied to acquire and analyse the data.

We found that 7.0 % or 10.1 % of the Actichip targets were not represented in the Affymetrix GeneChip or Operon array, respectively [see Additional file 2]. This result is not surprising considering that the three array platforms were implemented using different databases or different releases of the same database (Table 4) harbouring modifications of transcript sequences, identifiers or annotations. However, our data question the reliability of the high throughput design of pangenomic probe libraries. Focusing on a limited, easy-to-handle set of genes constitutes a more careful and robust approach. In line, several focused microarrays were recently described as powerful alternatives to whole genome arrays to study complex biological systems [45,51,52].

On the other hand, many of the genes represented on Actichip are highly similar and are not easy to discriminate using long oligonucleotide microarrays. When considering the actin gene family, only very limited regions of the transcript sequences can be used for the design of probes with convenient physical properties and specificity. To design high quality probes, we developed the CADO4MI program which allows a validation of oligonucleotides by cross-comparison of their sequences with data from several reference databases. For 219 of the 327 target genes represented on Actichip, combining information available from the UniGene and RefSeq databases actually allowed us to select probes with an enhanced specificity compared to those obtained using only one database. The fact that Actichip was able to differentiate the highly similar actin isoforms confirms that CADO4MI generates highly specific probes (Figure 4, Table 5). By contrast, some probes specific for the actin isoforms in the Affymetrix GeneChip and in the Operon set target regions having a high degree of similarity with several unrelated transcripts. As a consequence, these probes may generate false positive data due to cross-reactivity. This could explain the erroneous detections of some actin isoforms we observed with the Affymetrix or Operon platforms. In line, probe sequence alignment showed that the ACTA2 Operon probe has actually the potential to cross-hybridise with several transcripts [see Additional file 3]. By using the probe match tool at the NetAffx analysis center, we also found that the ACTA2 and ACTG1 probe sets from the U133A GeneChip both perfectly match with the ACTA2 mRNA. However, our data showed that the specificity of a probe can not be simply inferred from its design characteristics. Although giving false positives in our study, the ACTA1 Operon probe appeared to be specific as judged by sequence alignment [see Additional file 3], and the ACTG2 Affymetrix probe set perfectly matched with the corresponding transcript sequence.

It is conceivable that using latest versions of commercial arrays based on better-quality genome assembly and annotations or on new design concept may improve measurement accuracy and sensitivity. As an illustration, the GeneChip Exon array recently designed by Affymetrix with over six million probes targeting all annotated and predicted exons in the human genome appears as a promising tool to investigate both gene expression and alternative splicing with a high resolution. Data from the literature show that this chip may provide more accurate gene expression measurements than traditional microarrays [53,54], but requires a more complex strategy for the analysis of expression data [53,55]. Complex and time-consuming analysis is a typical trait of high densitiy microarrays and often represents the bottleneck of pangenomic expression studies. In the particular context of studies focusing on a limited number of genes, thematic arrays offer the possibility to overcome these limitations.

## Conclusion

Altogether, our data indicate that the tools and procedures we implemented in the course of our study constitute a powerful approach for the design of thematic arrays. Our data show that Actichip displays solid performance characteristics that make it a valid platform for functional genomics studies of the actin cytoskeleton in the context of basic or clinical research. Compared to high density microarrays, Actichip has the potential to facilitate gene expression data analysis because of its reasonable size. With the capacity to screen up to four samples in parallel, Actichip also contributes to lower the cost of the analysis.

## Methods

### Implementation of the Actinome database

Genomic data and sequence analysis features of the genes included in the Actichip microarray were compiled in the Actinome database [13]. To ensure robustness of the database, only mRNA sequences or complete coding cDNA sequences (CDS) were retrieved from the NCBI database, excluding expressed sequence tags (ESTs), sequence tagged sites (STS), genome sequence survey (GSS), working drafts and patent sequences. Each protein was annotated with high confidence through association with the corresponding full-length transcript and protein accession number using the RetScope platform, an in-house eukaryotic sequence analysis platform written in Tcl/Tk. Briefly, the protein accession numbers and sequences were first derived from the GenBank entries found by BLASTN homology searches from initial sequences [56]. The protein sequences were also inferred from the initial sequences by BLASTX searches in the UniProt database [57]. A double cross-validation was performed by assessing (*i*) sequence identity of the BLASTX- and BLASTN-derived proteins and (*ii*) their association with the same chromosomal localisation on the human genome [58]. As part of the RetScope platform, the GOAnno module [59] was finally used to annotate each protein entry with the corresponding Gene Ontology terms. GO annotations were retrieved using high quality multiple alignment of complete sequences computed for each protein.

### Oligonucleotide probe design

Oligonucleotide probes (60-mer) were designed using the program CADO4MI [24]. The program was written in Tcl/Tk and was successfully tested on various operating systems such as Windows (Microsoft), Solaris (Sun), Tru64UNIX (Compaq) and Linux (Fedora core 5, openSUSE 10.2). CADO4MI designed probes corresponding to a set of query sequences using different adjustable parameters through the following 4 steps [see Additional file 4] : (*i*) Poly(A) tails were masked in the query sequences (>15 consecutive adenosine bases), (*ii*) potential cross-hybridisations were detected using the entire sequence as a query in the BLASTN program [56], (*iii*) CADO4MI performed sequence analysis by moving iteratively (10 bases) over the query sequence a sliding window with a size corresponding to the oligonucleotide length (60-mer). At each iteration, sequences with no stretch of 7 or more contiguous identical bases, 35 %<GC content <70 %, 87°C< Tm < 97°C, and distance relative to 3'end ≤ 3.000 bases were selected. The melting temperature (Tm) was evaluated by the nearest-neighbor model combined with the unified parameters defined by SantaLucia [60]. The oligonucleotide specificity was checked through BLASTN by applying the Kane's rules to the selected sequences [14]. The process resulted in a list of oligonucleotides potentially targetting one or more sequences. (*iv*) The procedure was completed by selecting the oligonucleotide having the minimum number of non specific target and smallest distance to the 3' end of the query. Being a critical point in the design, the specificity of the probes was assessed automatically by identifying the complementary target sequences referenced in two nucleotide databases, RefSeq (release 1) and UniGene (build 161). Probes with unique targets in both databases were automatically selected by CADO4MI, whereas the others were curated manually.

### Actichip fabrication

Oligonucleotides were synthesized, 3'-end amino (C6)-modified and HPLC-purified by Eurogentec (Seraing, Belgium). Microarrays were manufactured by contact printing using a Microgrid II microarrayer equipped with 2500 split pins (Genomic solutions, Huntingdon, United Kingdom). Oligonucleotides were spotted in triplicate onto epoxide-coated glass slides (ArrayIt, Sunnyvale, CA, USA) at a concentration of 25 μM in microspotting plus solution (ArrayIt). The library was printed with two array patches per slide, each containing 32 human housekeeping genes as positive controls together with 41 sequences corresponding to viral or bacterial genes as negative controls. In addition, 10 spiking controls corresponding to *Arabidopsis thaliana *genes were incorporated in each array to assess the quality of microarray hybridisations as described below. Sequences of the corresponding 60-mer oligonucleotides were derived from those of 70-mer probes described elsewhere [30], and had no homology with any known human transcript sequence as assessed by a BLASTN analysis. Spotting was performed at a constant temperature of 22°C with 50% controlled humidity. Following arraying, the slides were dried overnight and were stored dessicated at room temperature. Actichip is available upon request [61].

### Cell culture and RNA sample preparation

Human breast adenocarcinoma MCF-7 cells (ATCC number HTB-22) were grown to 70–80 % confluency in Dulbecco's Modified Eagles's Medium (DMEM), 10 % Fetal Bovine Serum, 4 mM L-Glutamine, 100 units/ml penicillin G sodium and 100 μg/ml streptomycin sulfate. All cell culture reagents and buffers were purchased from Cambrex (Verviers, Belgium). Cells were washed twice with cold phosphate buffered-saline (PBS), and total RNA was extracted using the TRIzol reagent (Invitrogen, Merelbeke, Belgium) according to the manufacturer's intructions. A single batch of poly(A+) RNA was prepared from total RNA using the polyA purist kit from Ambion (Huntingdon, United Kingdom), and was stored frozen at -80°C in DEPC-treated water until use. Commercial poly(A+) RNA purified from human skeletal muscle was obtained from Ambion. RNA integrity and concentration were evaluated by the Agilent Bioanalyser 2100 capillary electrophoresis RNA 6000 nano assay (Agilent Biotechnologies, Diegem, Belgium). High quality RNAs with a ribosomal RNA ratio greater than 1.9 and no evidence of degradation were used in this study.

### Gene expression profiling experiments

#### Two-color microarrays

Gene profiling experiments were performed using procedures specific for either the Actichip microarray or the whole human genome array manufactured by the genomics laboratory at the university medical center of Utrecht (UMCU, The Netherlands) [see Additional file 5]. Briefly, 2 μg of poly(A+) RNA were reverse-transcribed using the Superscript II reverse transcriptase (Invitrogen) and were labeled with Alexa fluor 555 or 647 NHS-ester dyes (Invitrogen). The hybridisation was carried out at 42°C for 20 h in a Slidebooster 800 (Advalytix, Brunnthal, Germany) with a regular microagitation of the sample. Slides were scanned immediately after post-hybridisation washing using a Genepix 4000B microarray fluorescence reader (Molecular Devices, Sunnyvale, CA, USA) at a resolution of 10 μm. A percentage of saturated pixels of 0.1 was tolerated during the image acquisition to allow detection of the lowly expressed transcripts. Images were quantified using the Genepix Pro 6.0 software (Molecular Devices). For each spot, the local median background was subtracted from the median foreground signal. A spot was considered as positive (*i*) when it contained more than 55 % of foreground pixels above the background level + 1 standard deviation (STD) and (*ii*) less than 3 % of foreground pixels saturated, (*iii*) when the linear regression between the population of foreground pixels in the two channels computed using the least-squares method was above 0.5, and (*iv*) when the median of the pixel intensities at each wavelength with the median background pixel intensity at each wavelength subtracted was above 500. A positive feature was rated as relevant when the mean foreground intensity was above the mean foreground signal calculated from all negative control spots in at least one channel. Log_2 _ratio independency from signal intensity in both colour channels was assessed by displaying M-A plots (Log_2 _ratio = f(Log_2 _(median signal intensity at 532 nm × median intensity at 635 nm)/2). To compensate for dye bias, fluorescence data were subjected to a ratio-based, global normalisation considering the median intensity ratios of housekeeping genes. Genes detected in less than 2/3 of the arrays or exhibiting absolute Log_2 _ratios < 1 were filtered out. Clustering and graphical analysis of the remaining gene expression data were performed using the Acuity 4.0 software (Molecular Devices). Microarray data were in compliance with the standards proposed by the Microarray Gene Expression Data Society [62], and were deposited in the ArrayExpress public repository [63].

#### Single color microarray

Transcriptome analysis were carried out using the GeneChip HG-U133A 2.0 (Affymetrix, Santa Clara, CA, USA). Sample labeling, hybridisation and staining were performed at the Institut de Génétique et de Biologie Moléculaire et Cellulaire (IGBMC, Strasbourg, France) according to the eukaryotic target preparation protocol in the Affymetrix technical manual for Genechip expression analysis [see Additional file 5]. Briefly, 200 ng of purified poly(A+) RNA were linearly amplified to generate biotin-labeled cRNA. Upon fragmentation, labeled cRNA were hybridised for 16 h at 45°C according to the manufacturer's protocol. The hybridised arrays were washed and stained with streptavidin-phycoerythrin (Invitrogen), and signal was amplified with biotinylated anti-streptavidin antibodies (Sigma, Bornem, Belgium) using a Genechip fluidics station 400 (Affymetrix) according to the manufacturer's protocol. The arrays were scanned using a Genechip scanner 3000 (Affymetrix) at a resolution of 1.56 μm. The fluorescent intensity of each probe was quantified using the Microarray Analysis Suite version 5.0 (MAS 5.0) software (Affymetrix) according to manufacturer's instructions. The expression level of a single mRNA, defined as the signal, was determined by the MAS 5.0 software which uses a weighted average fluorescence intensity difference obtained among the 11 probe pairs that interrogate the expression of each individual gene. This software also makes a detection call (present [P], marginal [M], or absent [A]) for each gene or probe set, based on the consistency of the performance of the individual probe pairs, the hybridization above background, and the signal-to-noise ratio. Data analysis was performed using default parameters (Tau = 0.015).

### Significance analysis of microarray data

The significance level achieved with Actichip microarrays was evaluated by analysing the Log_2 _ratios from replicated experiments using the Significance Analysis of Microarrays algorithm (SAM; [31]) in Microsoft Excel (addin vs 2.21). SAM uses a modified t-test to determine for each gene represented on an array the relative difference in gene expression d(i), taking into account both the absolute level of expression as well as the standard deviation of the replicates. SAM then estimates the expected relative difference in expression de(i) for each gene by analysing permutations of the measurements. On a plot of de(i) *vs*. d(i), genes identified simply by chance are aligned on the d(i) = de(i) line whereas the genes potentially significant are represented by points displaced from this line by a distance greater than a threshold Δ. SAM also gives access to the percentage of genes found to be significant by chance, the false discovery rate (FDR).

### Actin probe specificity

#### PCR primer design

PCR primers (18-mer) were designed for the specific amplification of the six actin genes; actin, alpha 1, skeletal muscle (ACTA1, NM_001100), actin, alpha 2, smooth muscle, aorta (ACTA2, NM_001613), actin, beta (ACTB, NM_001101), actin, alpha, cardiac muscle 1 (ACTC1, NM_005159), actin, gamma 1 (ACTG1, NM_001614), actin, gamma 2, smooth muscle, enteric (ACTG2, NM_001615). For all isoforms except ACTA1, the forward primer was designed in a conserved region whereas the different reverse primers were specific for the respective actins [see Additional file 6]. All primers were verified by using the BLASTN program.

#### PCR

The PCR reaction was performed using as template cDNA generated from poly(A)RNA extracted from Hela cells. Reaction mixtures were prepared by using 10× *Ex Taq *reaction buffer and 2.5 U of *Ex Taq *polymerase (Takara Biomedicals, Otsu, Shiga, Japan) in accordance with manufacturer's recommendations in a total volume of 50 μl or 100 μl. Thermal cycling was carried out using a Robotcycler Gradient 96 (Stratagene, La Jolla, CA, USA) with an initial denaturation step of 94°C for 4 min, followed by 30 cycles of denaturation at 94°C for 30 s, an annealing step at 58°C for 30 s, and an elongation step at 72°C for 30 s. Cycling was completed by a final elongation step of 72°C for 10 min. The presence and size of the amplification products were determined by agarose (1%) gel electrophoresis of the reaction product.

#### Labeling and Hybridisation

Specific PCR products were labeled chemically using the ULYSIS labeling kit (Invitrogen) and Alexa Fluor 546 and 647 dyes according to the manufacturer's protocol. The labeled DNA was purified with Qiagen QIAquick PCR Purification kit, and was than recovered by ethanol precipitation, followed by resuspension in DIG Easy Hyb hybridization solution (Roche Diagnostics, Mannheim, Germany) at a concentration of 12.5 μg/ml. Hybridisations to Actichip microarrays were carried out for 16 h at 42°C as described previously using 20 μl of amplified and labeled DNA solution and a 22×25 mm LifterSlip. After incubation, microarrays were washed and scanned as described previously.

### Actichip sensitivity and detection limit

#### Preparation of spiked RNA samples

Spike poly(A+) RNAs were synthetised from the *Arabidopsis thaliana *spiking control cRNA vector set originally developed at the Institute for Genomic Research (TIGR, Rockville, MD, USA) by *Hind*III-*Sac*I directional cloning of PCR fragments corresponding to ten selected *A. thaliana *genes into the pSP64 poly(A) vector (Promega, Madison, WI, USA) [30]. Seven of these plasmids were linearised by *EcoR*I digestion, the restriction site being positioned immediately after the poly(A) tail sequence. One μg of each linearised plasmid was used as template for the *in vitro *synthesis of sense transcripts using the MEGAscript High Yield Transcription kit (Ambion). Following DNAseI treatment, the transcribed RNAs were purified by lithium chloride precipitation and resuspended in 10 mM Tris-HCl pH 7.5. The quality and quantity of the RNA samples were assessed with a RNA Labchip (Agilent Biotechnologies) and classical spectrophotometry. RNA solutions were adjusted at a concentration of 3 μg/μl corresponding to 10^6 ^spike copies/cell/μl (cpc/μl), and were mixed to prepare seven 10× test samples, each containing a full range of spike RNAs at concentration ranging from 1 to 10^5 ^cpc [see Additional file 1]. Transcript copy number calculations were made assuming that a cell contains 1 pg poly(A) RNA corresponding to an average of 360,000 transcripts, and that 0.3 ng spike transcript corresponds to 100 spike copies/cell. Care was taken to use DEPC-treated water containing 1 μg/μl *E. coli *tRNA (Roche Diagnostics) to prevent the loss of spike RNAs at low concentrations through adsorption on plastic surfaces. An eighth 10 × RNA sample was constructed containing the seven RNA spikes at a concentration corresponding to 10^3 ^cpc.

#### Dose response curves

Dose response curves were determined using a procedure modified from Allemeersch et al. [41]. Briefly, the seven test mixes containing the spike RNAs in well-defined concentration and expression ratio were used as template to prepare the Alexa dye 647-labeled samples, whereas the reference mix was used for the generation of the Alexa fluor 555-labeled sample. Test and reference samples were pooled in equimolar concentration to obtain seven hybridisation mixtures that were incubated for 20 h at 42°C onto distinct Actichip microarrays. Slides were washed and scanned as described previously.

### Microarray platform comparison

Sets of common probes between the different microarray platforms were established on the basis of sequence accession numbers and/or sequence comparison. In the first approach, RefSeq or GenBank accession numbers from Actinome sequences were compared to the accession numbers present in the annotation files provided by the UMCU for the Operon probe set and by Affymetrix for the HG-U133 2.0 GeneChip. In the second approach, sequences of the Actichip targets (GenBank mRNA sequences) were compared to those provided by Affymetrix ("target sequence") and the UMCU (GenBank and RefSeq mRNA sequences). For this sequence similarity search, we used the BLASTN program and the calculation of two parameters, a global percent identity (GID) and a percent coverage (pCover). GID was defined as the ratio of the number of identical residues to the total number of residues in all Maximum Segment Pairs (MSPs) of the query [see Additional file 7]. pCover corresponded to the ratio of the number of identical residues to the number of residues that were aligned between the two sequences. To avoid false negative results, similarity searches were performed using permissive cutoffs of 95% and 70 % for GID and pCover, respectively. All alignments were then curated manually.

## Authors' contributions

JM, FC and ArM developed CADO4MI and processed the bioinformatics data. JM also contributed to data evaluation and drafted the manuscript. AnM and GV contributed to protocol optimization and some experiments as well as data evaluation. MY gave statistics support, contributed to data evaluation and helped to draft the manuscript. OP, EF and LV conceived the study, participated in its design and coordination. LV developed optimized protocols, prepared the Actichip microarrays, carried out microarray experiments, contributed to oligonucleotide design and data evaluation and drafted the manuscript. All authors read and approved the final manuscript.

## Supplementary Material

Additional file 1 Concentration of the spike RNAs in the seven 10× sample mixes and 10× reference mix. Concentration is expressed in copies per cell (cpc). The gene abbreviations correspond to those used in the original description of the *A. thaliana *control set [30]. Click here for file

Additional file 2 Gene coverage of the Actichip, Affymetrix and Operon platforms. The list recapitulates the genes included in the Actichip microarray that were not covered by the Affymetrix HG-U133A 2.0 GeneChip and the Human oligonucleotide set 2.0 from Operon. Data relative to the Affymetrix GeneChip were verified at the NetAffx analysis center. Click here for file

Additional file 3 Probe design comparison. The sequences of the probes or probe sets specific for the various actin isoforms in the Actichip, Operon and Affymetrix platforms were aligned with the sequence of the corresponding target. The results are displayed in the graphical user interface of CADO4MI. The top panel shows the evolution of the average percent identity (red curve) and the number of sequence detected by BLASTN (blue curve) along the sequence of the target (red rectangle). The bottom panel displays the position of the probes relative to the sequence of the query. The large dark gray rectangle corresponds to the target sequence used by Affymetrix to design the probe sets. Click here for file

Additional file 4 CADO4MI user manual. Click here for file

Additional file 5 Experimental protocols for transcriptome analysis. Click here for file

Additional file 6 Primers used for PCR amplification of the actin isoforms. Click here for file

Additional file 7 GID and pCover parameters. Sequences of the Actichip targets (GenBank mRNA sequences) were compared to the sequences (GenBank and RefSeq mRNA sequences) provided by Affymetrix ("target sequence") and the genomics laboratory at the university medical center of Utrecht (UMCU, The Netherlands) relative to the Operon probe set. This comparison was performed through sequence alignment using the BLASTN program and calculation of two parameters, a global percent identity (GID) and a percent coverage (pCover). GID corresponds to the percentage of global identity between the query sequence (Qseq; Affymetrix or Operon target sequence) and one sequence (SeqB; Actichip target sequence) in the BLAST output. GID is determined by considering all best scoring alignments (MSP) between QSeq and SeqB and is computed as the ratio of the total number of identical residues (TNAR) to the total number of residues (TNRM) in all these alignments. pCover refers to the percentage of sequence coverage between QSeq and SeqB in the BLAST output. The coverage corresponds to the extended overlapping regions between QSeq and SeqB considering all the regions aligned between the two sequences. pCover is computed as the ratio of the sum of all identical residues (NAR) to the number of residues in coverage (NRC). Click here for file
