# Supplementary material for: Design and evaluation of Actichip, a thematic microarray for the study of the actin cytoskeleton
Source: BMC Genomics. 2007 Aug 29;8:294. doi: 10.1186/1471-2164-8-294 (PMC2077341; doi:10.1186/1471-2164-8-294)
Supplement: Additional file 2 — Gene coverage of the Actichip, Affymetrix and Operon platforms. The list recapitulates the genes included in the Actichip microarray that were not covered by the Affymetrix HG-U133A 2.0 GeneChip and the Human oligonucleotide set 2.0 from Operon. Data relative to the Affymetrix GeneChip were verified at the NetAffx analysis center. [file 1471-2164-8-294-S2.pdf]

Microarray Platform	Gene name	GenBank Accession Number	RefSeq Accession Number	Uniprot ID Accession Number	Definition
Human genome oligonucleotide set, version 2 (Operon)	RPSA	BC005391	NM_002295	P08865	40S ribosomal protein SA (P40) (34/67 kDa laminin receptor) (Colon carcinoma laminin-binding protein) (NEM/1CHD4) (Multidrug resistance- associated protein MGr1-Ag).
	ACTC1	BC009978	NM_005159	P04270	Actin, alpha, cardiac muscle 1 (ACTC1).
	ANK2	X56958	NM_020977	Q01484	Ankyrin 2 (Brain ankyrin) (Ankyrin B) (Ankyrin, nonerythroid).
	ANXA2	BC001388	NM_004039	AAH52567	Annexin A2 (Annexin II) (Lipocortin II) (Calpactin I heavy chain) (Chromobindin 8) (P36) (Protein I) (Placental anticoagulant protein IV) (PAP-IV).
	DST	AF400226	NM_015548	O94833	Bullous pemphigoid antigen 1, isoforms 6/9/10 (Trabeculin-beta) (Bullous pemphigoid antigen) (BPA) (Hemidesmosomal plaque protein) (Dystonia musculorum protein).
	CLMN	AB047979	NM_024734	Q96JQ2	Calmin (calponin-like, transmembrane).
	CDC42	M57298	NM_001791	P21181	Cell division control protein 42 homolog (G25K GTP-binding protein).
	DAAM1	AK093813	NM_014992	Q8N1Z8	Dishevelled associated activator of morphogenesis 1 (DAAM1).
	DMD	BC028720	NM_004016	Q8N754	Dystrophin (Muscular dystrophy, Duchenne and Becker types).
	EEF1A1	AF267861	NM_001402	Q9H2I7	EF1a-like protein.
	EEF1G	AF119850	NM_001404	AAH28179	Eukaryotic translation elongation factor 1 gamma (EEF1G).
	EVL	BC023997	NM_016337	Q9UI08	Ena/vasodilator stimulated phosphoprotein-like protein (Ena/VASP-like protein).
	FN1	AJ535086	NM_054034	P02751	Fibronectin precursor (FN) (Cold-insoluble globulin) (CIG).
	GAS2	BC040470	NM_177553	O43903	Growth-arrest-specific protein 2 (GAS-2).
	DSG4	BC039098	NM_177986	ACT0999	Desmoglein 4, mRNA (cDNA clone IMAGE:4822945), partial cds.
	SSH1	AB072355	NM_018984	Q8WYL5	Slingshot homolog 1 (Drosophila) (SSH1).
	SSH2	AB072358	NM_033389	Q8WYL2	Slingshot homolog 2 (Drosophila) (SSH2).
	BIN1	AL713697	NM_139343	CAD28496	Bridging integrator 1 (BIN1).
	TPM3	AK092690	NM_152263	Q8NAH0	Tropomyosin 3 (TPM3).
	ITGB1	X07979	NM_002211	P05556	Integrin beta-1 precursor (Fibronectin receptor beta subunit) (CD29 antigen) (Integrin VLA-4 beta subunit).
	JUP	BC011865	NM_021991	Q9BWC4	Junction plakoglobin.
	KRT8	X74929	NM_002273	P05787	Keratin, type II cytoskeletal 8 (Cytokeratin 8) (K8) (CK 8).
	LSP1	BC001785	NM_002339	P33241	Lymphocyte-specific protein 1 (Protein pp52) (52 kDa phosphoprotein) (Lymphocyte-specific antigen WP34) (47 kDa actin binding protein).
	MACF1	AF141968	NM_012090	Q9UPN3	Microtubule-actin crosslinking factor 1, isoforms 1/2/3 (Actin cross- linking family protein 7) (Macrophin 1) (Trabeculin-alpha) (620 kDa actin-binding protein) (ABP620).
	MYO1F	AK092877	NM_012335	O00160	Myosin If (Myosin-IE).
	NRAP	AY081943	NM_006175	Q8TCH0	Nebulin-related anchoring protein.
	SYNE1	AF495910	NM_033071	Q8NF91	Nesprin 1 (Nuclear envelope spectrin repeat protein 1) (Synaptic nuclear envelope protein 1) (Syne-1) (Myocyte nuclear envelope protein 1) (Myne-1) (Enaptin).
	PDCD6IP	BC020066	NM_013374	Q8WUM4	Programmed cell death 6 interacting protein (ALG-2 interacting protein 1) (Hp95).

	CFLP1	BC031631	NM_152427	Q8N1B5	Similar to cofilin, non-muscle isoform (18 kDa phosphoprotein) (P18).
	TLN2	AF402000	NM_015059	Q9Y4G6	Talin 2.
	TMSB4X	BC016732	NM_021109	CAC43317	Thymosin beta-4.
	TNNT2	X74819	NM_000364	P45379	Troponin T, cardiac muscle isoforms (TnTC) (cTnT).
	TMOD3	BC020542	NM_014547	Q9NYL9	Ubiquitous tropomodulin (U-Tmod) (Tropomodulin 3).
GeneChip HG-U133A 2.0 (Affymetrix)	ANLN	AF273437	NM_018685	Q9NQW6	Actin binding protein anillin.
	ACTRT2	AF440740	NM_080431	Q8TDG1	Actin-related protein T2.
	SCIN	BC021090	NM_033128	Q9Y6U3	Adseverin (Scinderin).
	ANGPTL6	AB054064	NM_031917	Q8NI99	ARP3.
	AXIN2	AF078165	NM_004655	Q9Y2T1	Axin 2 (Axis inhibition protein 2) (Conductin) (Axin-like protein) (Axil).
	ADD2	X58199	NM_001617	P35612	Adducin 2 (beta) (ADD2) (Beta adducin) (Erythrocyte adducin beta subunit).
	DBNL	AF151364	NM_014063	Q9NR72	Cervical SH3P7 (Mucin-associated protein).
	CFL2	AF134802	NM_021914	Q9Y281	Cofilin, muscle isoform (Cofilin 2).
	CTTNBP2	AF377960	NM_033427	Q8WZ74	Cortactin-binding protein 2 (Hypothetical protein KIAA1758).
	NKD1	BC051288	NM_033119	Q969G9	Dvl-binding protein NKD1 (Naked cuticle-1) (Naked protein).
	PARVG	BC034406	NM_022141	Q9HBI0	Gamma-parvin.
	ARPM1	AK055346	NM_032487	Q96NJ0	Actin-related protein M1 (ARPM1)
	ACTRT1	BC014597	NM_138289	Q96L10	Actin-related protein T1 (ACTRT1)
	DSG4	BC039098	NM_177986	ACT0999	Desmoglein 4, mRNA (cDNA clone IMAGE:4822945), partial cds.
	SSH1	AB072355	NM_018984	Q8WYL5	Slingshot homolog 1 (Drosophila) (SSH1).
	SSH2	AB072358	NM_033389	Q8WYL2	Slingshot homolog 2 (Drosophila) (SSH2).
	ESPN	AL136880	NM_031475	Q9H0A2	Espin (ESPN).
	LAMA5	ACT0412	NM_005560	O15230	Laminin alpha-5 chain precursor.
	MYO1C	X98507	NM_033375	O00159	Myosin Ic (Myosin I beta) (MMI-beta) (MMIb).
	RHOJ	AK027351	NM_020663	Q9H4E5	Rho-related GTP-binding protein RhoJ (Tc10-like GTP-binding protein TCL).
	CFLP1	BC031631	NM_152427	Q8N1B5	Similar to cofilin, non-muscle isoform (18 kDa phosphoprotein) (P18).
	TMOD4	AF177173	NM_013353	Q9NZQ9	Skeletal muscle tropomodulin (Sk-Tmod) (Tropomodulin 4).
	TLN2	AF402000	NM_015059	Q9Y4G6	Talin 2.
