# Supplementary material for: Design and evaluation of Actichip, a thematic microarray for the study of the actin cytoskeleton
Source: BMC Genomics. 2007 Aug 29;8:294. doi: 10.1186/1471-2164-8-294 (PMC2077341; doi:10.1186/1471-2164-8-294)
Supplement: Additional file 3 — Probe design comparison. The sequences of the probes or probe sets specific for the various actin isoforms in the Actichip, Operon and Affymetrix platforms were aligned with the sequence of the corresponding target. The results are displayed in the graphical user interface of CADO4MI. The top panel shows the evolution of the average percent identity (red curve) and the number of sequence detected by BLASTN (blue curve) along the sequence of the target (red rectangle). The bottom panel displays the position of the probes relative to the sequence of the query. The large dark gray rectangle corresponds to the target sequence used by Affymetrix to design the probe sets. [file 1471-2164-8-294-S3.pdf]

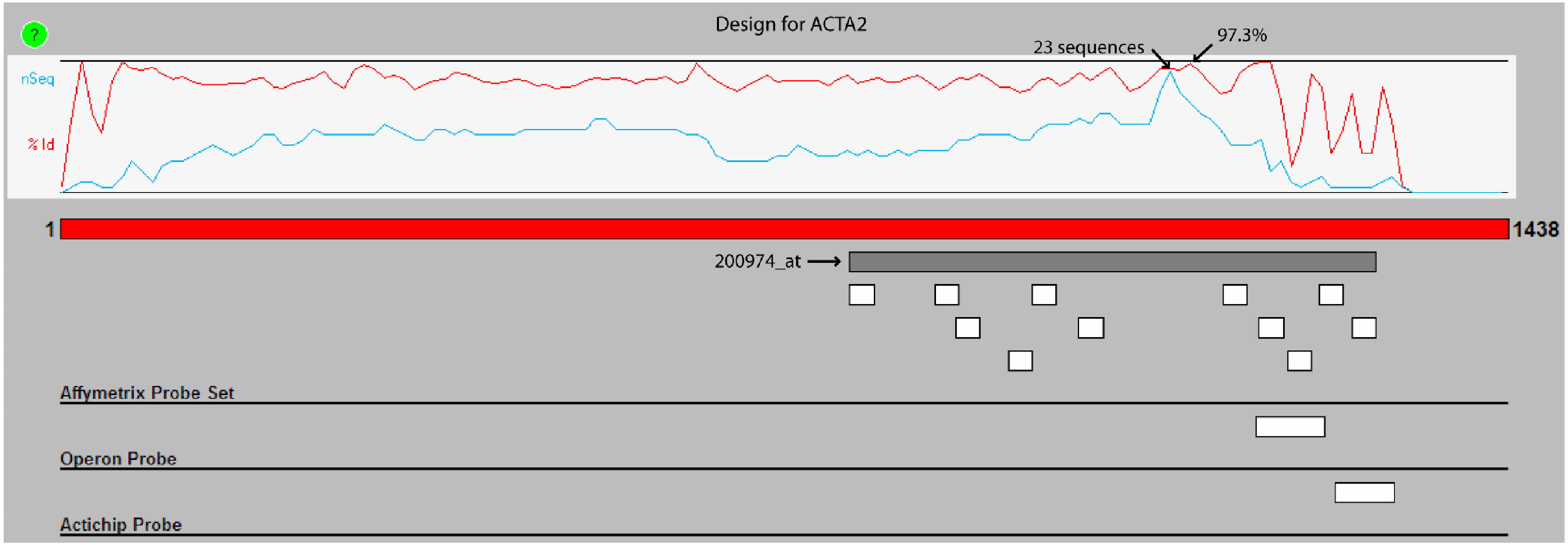
Design for ACTA2
23 sequences
97.3%
nSeq
% Id
1
1438
200974_at
Affymetrix Probe Set
Operon Probe
Actichip Probe

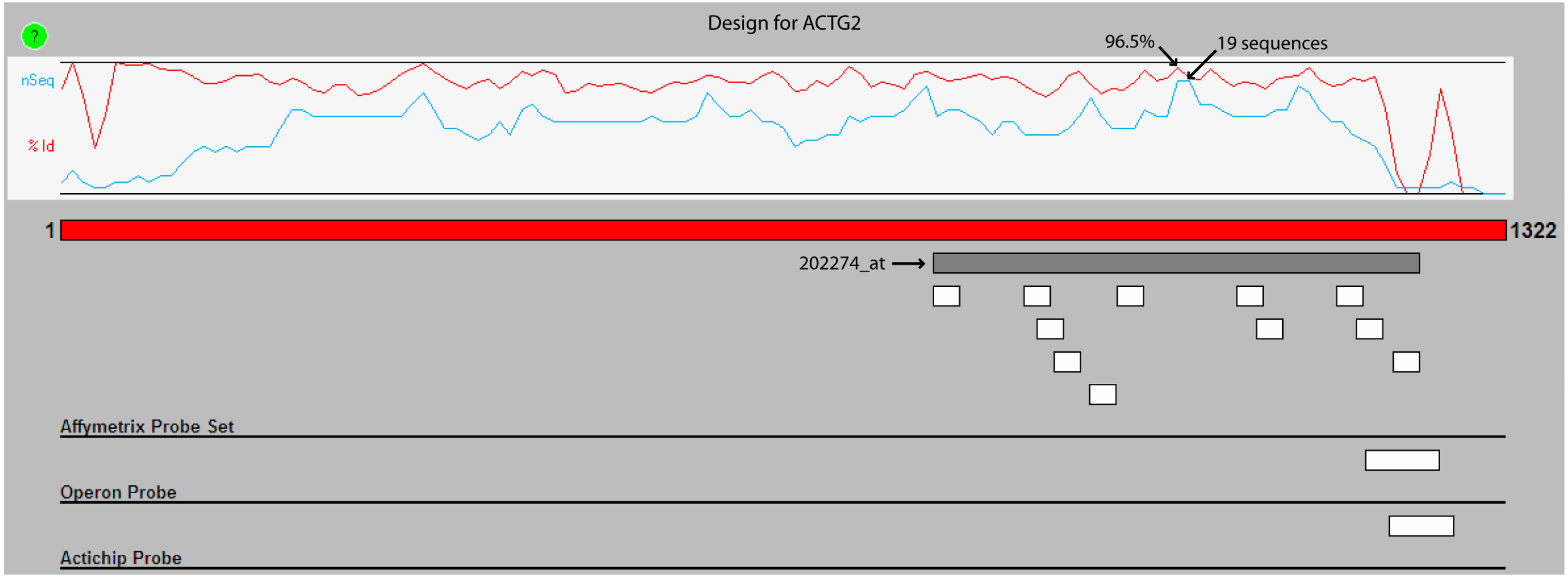

Design for ACTG2
96.5%
19 sequences
nSeq
% Id
1
1322
202274_at
Affymetrix Probe Set
Operon Probe
Actichip Probe

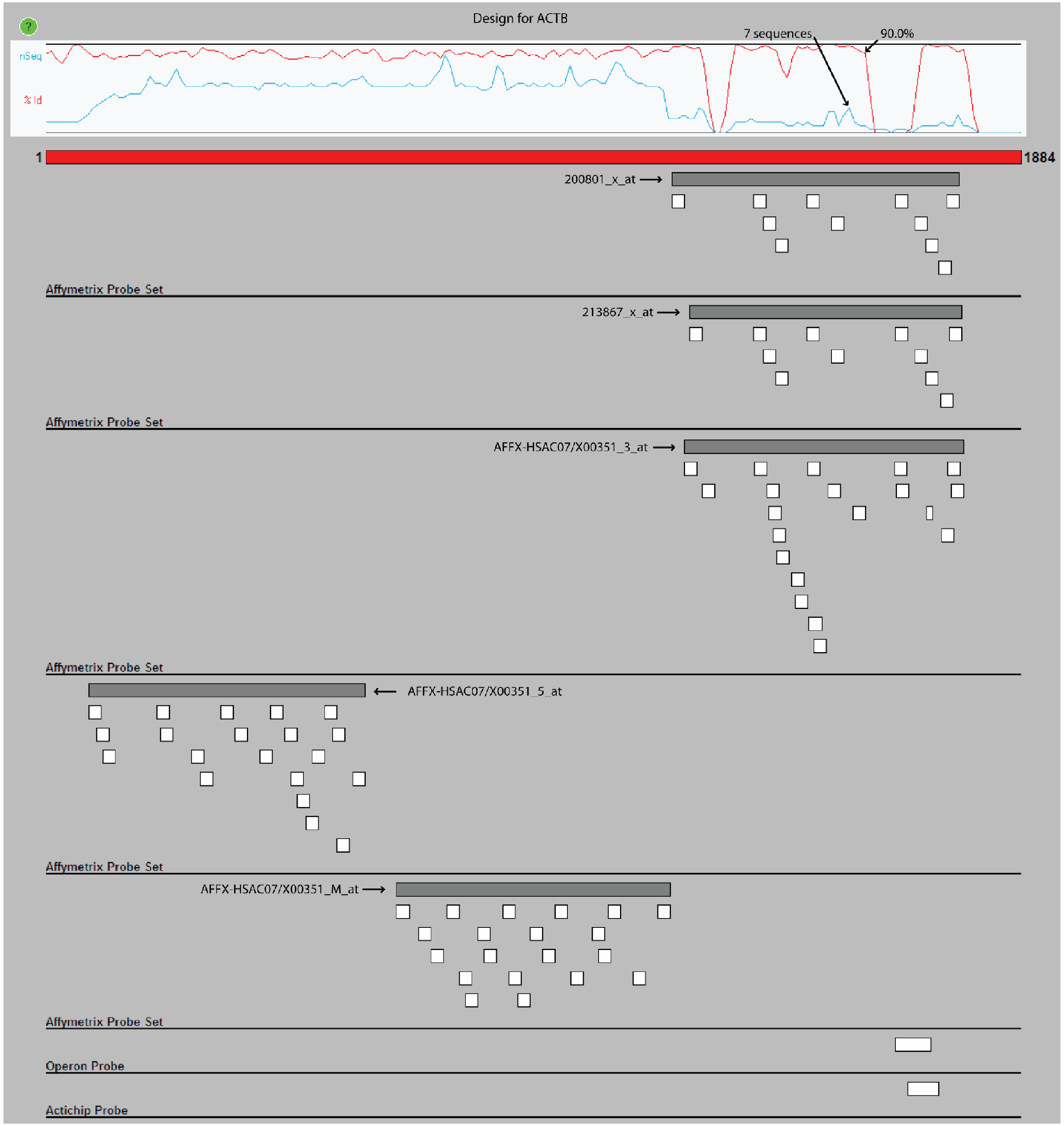

Design for ACTB
7 sequences
90.0%
nSeq
% Id
1
1884
200801_x_at
Affymetrix Probe Set
213867_x_at
Affymetrix Probe Set
AFFX-HSAC07/X00351_3_at
Affymetrix Probe Set
AFFX-HSAC07/X00351_5_at
Affymetrix Probe Set
AFFX-HSAC07/X00351_M_at
Affymetrix Probe Set
Operon Probe
Actichip Probe

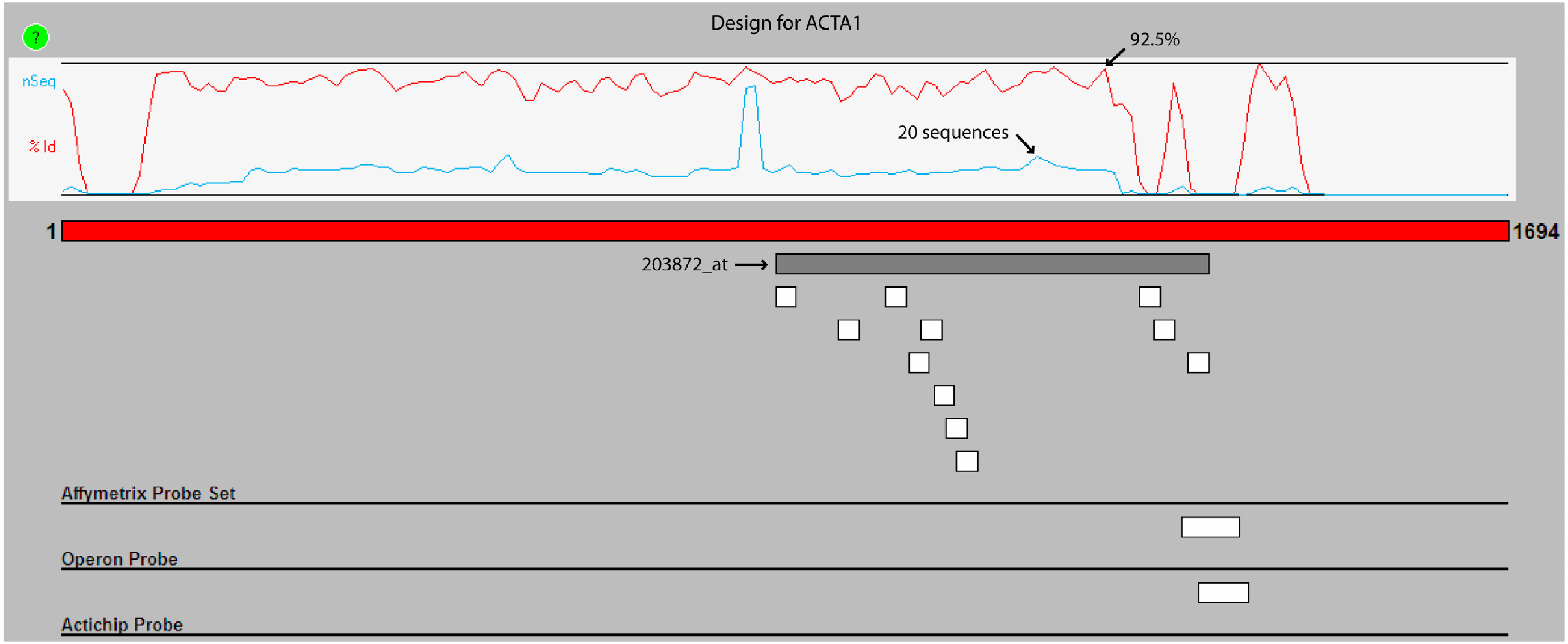
Design for ACTA1
92.5%
nSeq
% Id
20 sequences
1
1694
203872_at
Affymetrix Probe Set
Operon Probe
Actichip Probe

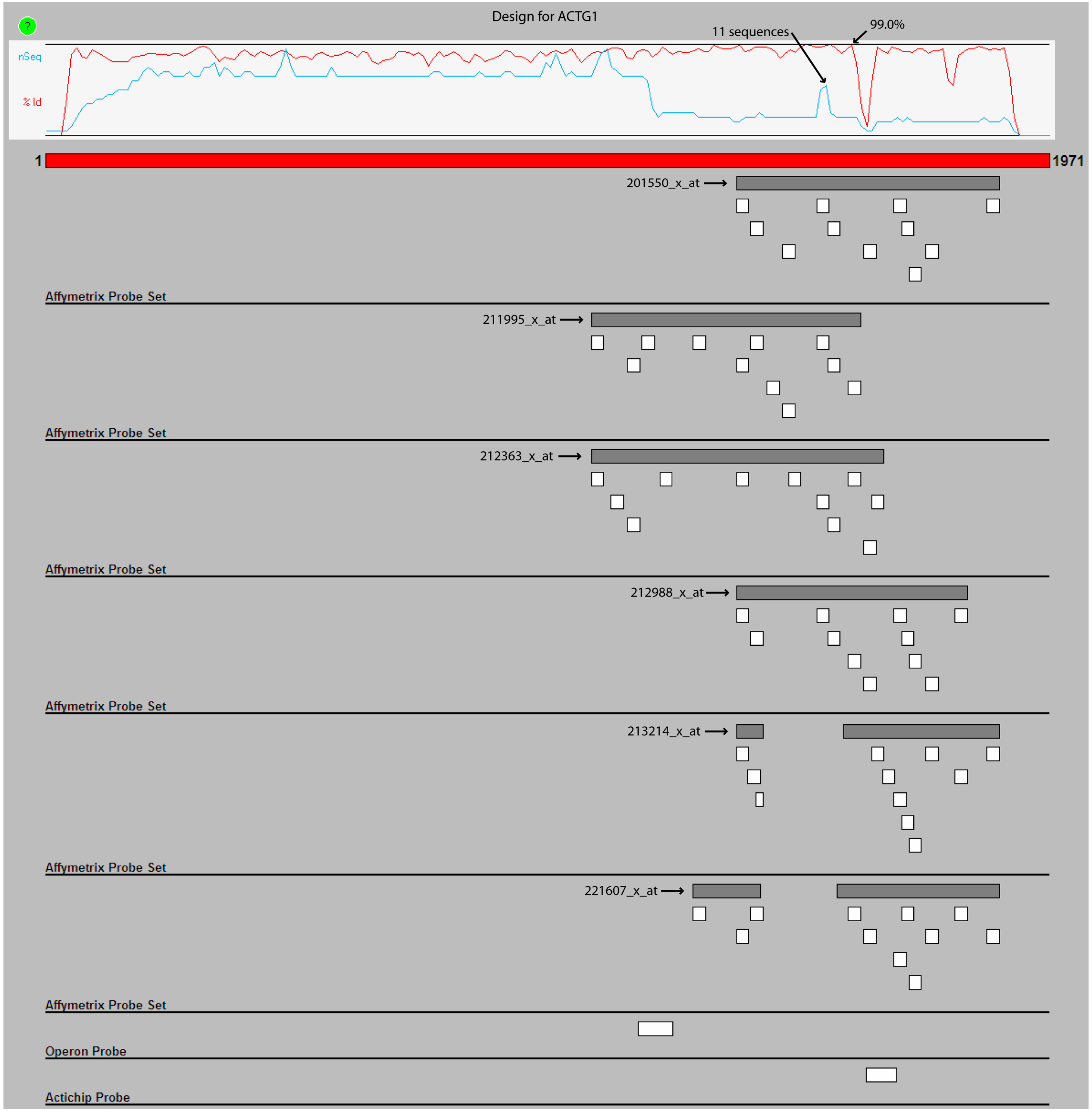
Design for ACTG1
11 sequences
99.0%
nSeq
% Id
1
1971
201550_x_at
Affymetrix Probe Set
211995_x_at
Affymetrix Probe Set
212363_x_at
Affymetrix Probe Set
212988_x_at
Affymetrix Probe Set
213214_x_at
Affymetrix Probe Set
221607_x_at
Affymetrix Probe Set
Operon Probe
Actichip Probe

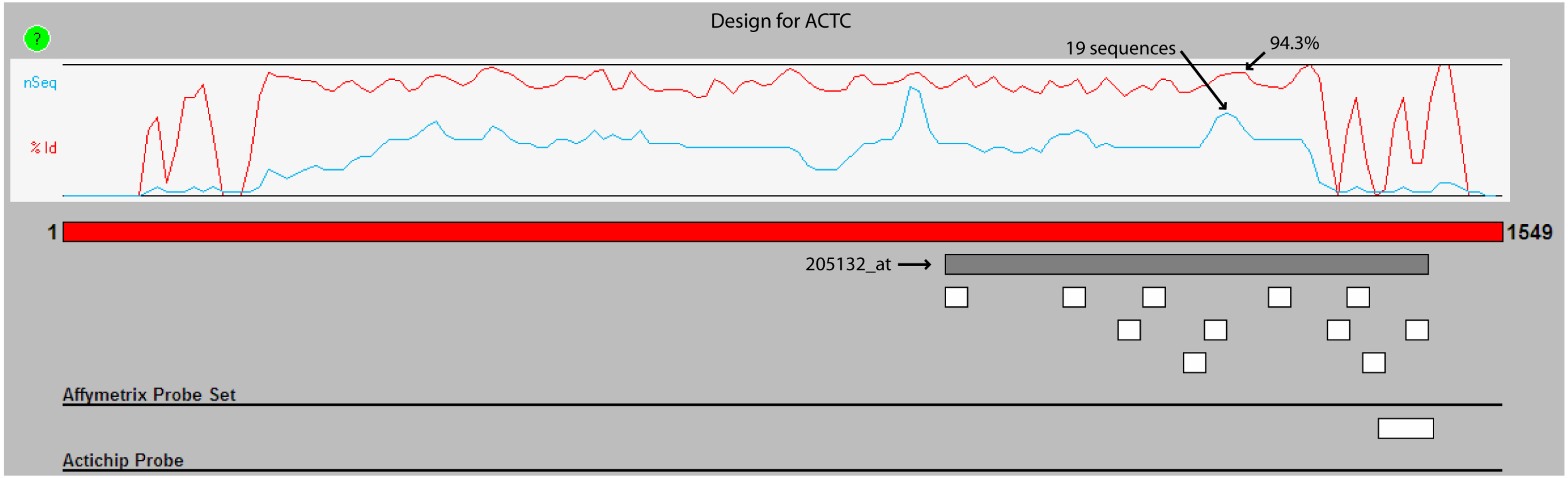
Design for ACTC
19 sequences
94.3%
nSeq
% Id
1
1549
205132_at
Affymetrix Probe Set
Actichip Probe
