# Supplementary material for: Design and evaluation of Actichip, a thematic microarray for the study of the actin cytoskeleton
Source: BMC Genomics. 2007 Aug 29;8:294. doi: 10.1186/1471-2164-8-294 (PMC2077341; doi:10.1186/1471-2164-8-294)
Supplement: Additional file 4 — CADO4MI user manual. [file 1471-2164-8-294-S4.pdf]

CADO4MI

Computer Assisted Design of Oligonucleotide For Microarray

1.5

Documentation

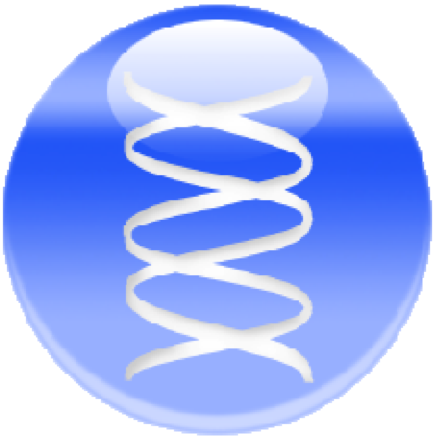

TABLE OF CONTENTS

1. General 3
2. Installation 3
   a. Tcl/Tk 3
   b. BLAST 3
   c. CADO4MI 4
3. Principles 4
   a. Design of oligonucleotides 4
   b. Transcript sequences 4
   c. Specificity 4
   d. Melting temperature 5
      (1) Nearest Neighbor model 5
      (2) Wallace method 5
      (3) Long oligonucleotide methods 5
   e. CADO4MI Protocol 6
      (1) Masking sequence 7
      (2) BLASTN 7
      (3) Sequence analysis 7
      (4) Best oligonucleotide selection 8
   f. Input and Output 8
      (1) BLAST database 8
      (2) Input 9
      (3) Output 9
4. Graphical User Interface (GUI) 10
   a. Start Panel 11
   b. Oligo Design Menu 12
      (1) Parameters Panel 12
      (2) Advanced Parameters 13
      (3) Batch Panel 13
   c. Design Result Menu 14
      (1) Interactive board 15
      (2) Design board 15
      (3) Buttons Board 19
5. Command line 21
6. Appendix 22
   Fasta Format 22
   Sequence Validation. 23
   Information Panel 24
   Graph Panel 27
   Parameter estimation 29
   Blast tools 32
7. References 35
8. FAQ 36

1. General

CADO4MI is a program for automatic design of oligonucleotides, fully customizable and accessible through a graphical user interface. For an input set of sequences, it allows parameter optimization and can combine multiple designs in distinct sequence databases. CADO4MI is distributed using the GNU GPL License.

2. Installation

CADO4MI is written in Tcl/Tk and runs on all platforms with a standard Tcl/Tk 8.4 installation and the BLAST program installed (Altschul, et al., 1997). It has been successfully tested on Windows (Microsoft), Solaris (Sun), Tru64 UNIX (Compaq), and Linux (Fedora core 5, OpenSuse 10.2).

a. Tcl/Tk

The Tcl/Tk plug-in can be downloaded for any architecture at the ActiveState web site http://www.activestate.com/Products/ActiveTcl. Simply download and install. On Unix check where the wish binary is located. CADO4MI assume it is in /usr/bin/wish. If this is not the case, simply move the binary to that location or change the first line in CADO4MI.tcl according to your system.

b. BLAST

CADO4MI requires also the BLAST program to search for sequence similarities. It can be downloaded from the NCBI web site using the following link (http://www.ncbi.nlm.nih.gov/blast/download.shtml). Install and make sure that blastall (and fastacmd) program (blastall binary directory) is available in your PATH environment variable. You can also define the BLASTDB environment variable and CADO4MI will use it to search for BLAST databases (this is an option).

Under Windows system, to manage environment variables, you will need to open **Control Panel-Performance and Maintenance-System** (or right-click on **My Computer** and choose "Properties"). In the box that opens, click the "Advanced" tab to obtain a dialog box and click the button "Environment Variables". Then you can see if the desired variable exists and if not, create it with the button “New” or modify it with the button “Edit”. For the PATH variable which should already exist, be sure to remember to separate directory names with a semicolon.

Under Unix systems depending on the shell you can use for examples (“csh” shell):

```
printenv   BLASTDB                  - display BLASTDB value if it exists
setenv     BLASTDB /bioinfo/blast   - defines the blastdb to /bioinfo/blast
setenv     PATH /blast2.13:$PATH    - add /blast2.13 to the current PATH variable
```

If you have any problem with this you can also edit the file CADO4MI.path (directory of CADO4MI) and directly enter the PATH of the program. Be careful this file is a tab-

separated file using “#” as comments and the first word corresponds to the program name (no need to change) and the second (after a tab) to the program path:

```
Ex:  #PROGRAM PATH
     blastall     /usr/bioinf/blastall
     fastacmd     D:\Homology_Search\blast\fastacmd
```

c. CADO4MI

CADO4MI sources are available at http://bips.u-strasbg.fr/CADO4MI. Download and extract it in any directory. To launch CADO4MI, execute or double click the CADO4MI.tcl file. For convenience, you can make a link to this file (Windows or UNIX).

3. Principles

a. Design of oligonucleotides

The design of oligonucleotides is a cornerstone of modern molecular biology and has various critical applications (e.g. PCR, Southern blotting, microarray…). Among these, the microarray technology has become a standard tool allowing biologists to monitor simultaneously the expression level of thousands of genes. An oligonucleotide set should contain sequences specific to their target genes which minimize cross-hybridization signals (i.e. signals of non-target genes), close to the 3' end of transcripts sequence, with homogeneous melting temperature (T_m) and GC content to guarantee uniform hybridization.

b. Transcript sequences

Transcript sequences are often poly-A mRNA and can originate from various sources. These nucleotide sequences can be either absent or partially present in the sequence databases used to design the oligonucleotides and consequently influence this process. Therefore, CADO4MI includes a sequence validation module to warn the user of such situation (Appendix Sequence Validation).

c. Specificity

The specificity of the oligonucleotide sequences is defined by its ability to hybridize only to its target sequence. The cross-hybridization refers to the detection of non-target sequences by this oligonucleotide. This kind of signal can be avoided by excluding non-target sequences which have excess of sequence identity with the oligonucleotide (Kane, et al., 2000). The limits of cross-hybridizations have been experimentally determined for 50mers by Kane et al (Kane, et al., 2000) and similarly for 60mers by Hughes et al (Hughes, et al., 2001), as two rules (**Kane's rule**):

(1) Global percent identity should be < 75-80% with the target sequence.
(2) Contiguous stretches of identity with non target sequences should be less than 15 nts.

d. Melting temperature

The melting temperature (T_m) of a sequence is the temperature at which 50% of this sequence and its perfect complement are in duplex. The calculation of T_m is an important point in the design of oligonucleotide. Even though, the T_m calculations for microarray are only approximation due to the absence of model for fixed oligonucleotides, the important point is to use the same method (all oligonucleotides will be treated equally) and to design a set of oligonucleotides with a homogenous range of T_m values. This will guarantee the homogeneity of the hybridization step during microarray experiment. CADO4MI allows the user to select several methods for T_m calculation:

(1) Nearest Neighbor model

The default method is the nearest-neighbor thermodynamic model combined with the unified parameters defined by SantaLucia (Breslauer, et al., 1986; Rychlik, et al., 1990; SantaLucia, 1998). This model is suitable for sequences <70 bases.

$$T_m = \frac{1000 \times \Delta H}{\Delta S + R \times \ln\left(\frac{[DNA]}{4}\right)} - 273.5$$

Where:

R	=	Molar gas constant (1.9872 cal/K.mol)
H	=	Enthalpy for helix formation
S	=	Entropy for helix formation
DNA	=	DNA concentration (default 10e-6 M)

(2) Wallace method

Another widely used method to calculate T_m is known as the "Wallace method" (Wallace, et al., 1979) and is designed for short sequences (<30 bases). This method is often used for PCR primer design.

$$T_m = 2 \times (A + T) + 4 \times (G + C)$$

(3) Long oligonucleotide methods

Other methods designed for longer sequences exist with some variations (Bodkin and Knudson, 1985; Bolton and Mc, 1962; Casey and Davidson, 1977; Howley, et al., 1979; Meinkoth and Wahl, 1984):

- DNA-DNA

$$T_m = 81.5 + 16.6 \times (\log M) + 41 \times (\%GC) - 0.62(\%F) - 500/S$$

- DNA-RNA

$$T_m = 79.8 + 18.5 \times (\log M) + 58.4 \times (\%GC) + 11.8 \times (\%GC)^2 - 0.50(\%F) - 820/S$$

- RNA-RNA

$$T_m = 79.8 + 18.5 \times (\log M) + 58.4 \times (\%GC) + 11.8 \times (\%GC)^2 - 0.35(\%F) - 820/S$$

Where

M	=	Molar salt concentration (default 1 M)
GC	=	GC content
F	=	Percent of formamide (default 12.3%)
S	=	Size of the sequence

e. CADO4MI Protocol

The oligonucleotide design protocol is divided into 4 steps. The following figure illustrates the general flow chart within CADO4MI:

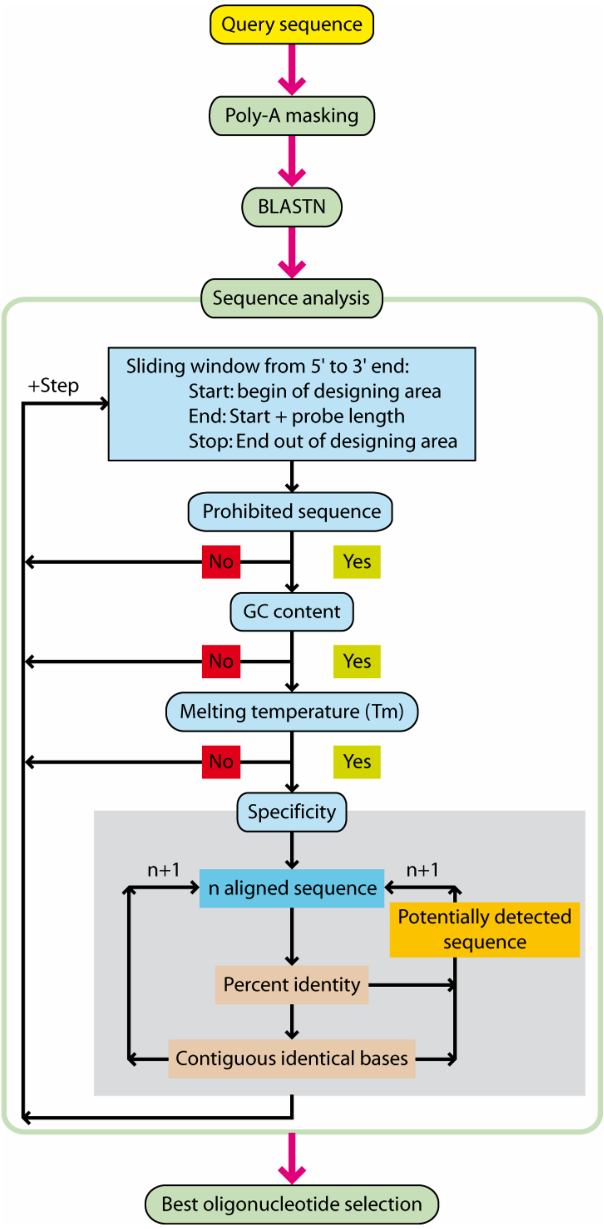

(1) Masking sequence

This step is set to remove the poly-A tail from mRNA. Indeed, poly-A tail is useful to separate mRNAs from other nucleic acids in molecular fraction but in our purpose this is a part of the mRNA sequence where no interesting oligonucleotide can be designed. Therefore, CADO4MI removes poly-A tail with a minimum length of 15 A.

(2) BLASTN

The alignment between input sequences and databases are done by the BLASTN program. Critical BLASTN parameters can be changed by the user but default exists. The BLASTN searches are computed using the complete query sequence.

(3) Sequence analysis

The analysis is done sequentially by moving a sliding window over the query sequence. The window size corresponds to the length of the oligonucleotide to design and move for 10 nucleotides (default parameter).

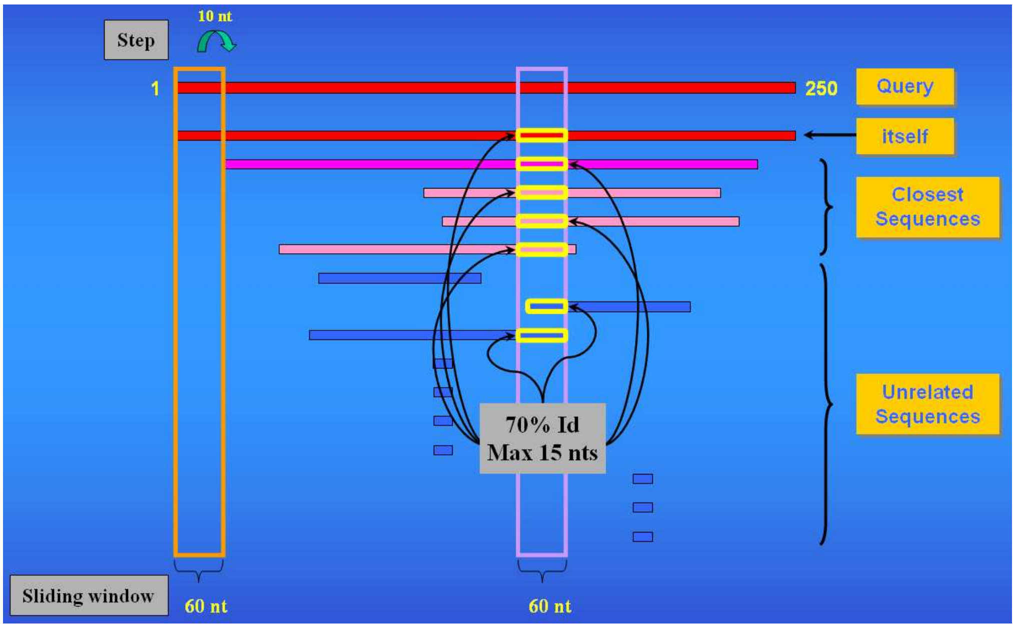

The program first determines the sequences areas where the design is requested and then every window sequence is analyzed according to the different criteria:

- First, window sequence containing prohibited sequences (non desired user defined sequences), undetermined or degenerated nucleotides is rejected from the analysis.
- Second, GC content is compared to threshold and out ranged sequence is excluded.
- Third, the T_m is estimated and out ranged sequence is excluded.
- Fourth, the specificity of each window sequence is filtered according to Kane’s rules. Therefore, the alignment is analyzed by calculating the overall percent identity and maximum number of identical contiguous bases of each potential candidate detected at this position in the BLAST result. The result of this step is a list of oligonucleotides potentially detecting 0, 1 or more sequences.

(4) Best oligonucleotide selection

The best oligonucleotide is then selected among all potential candidates using the number of potential detected sequences and the position in the query as criteria. We choose the oligonucleotides with less X-hybridization sequences and the closer to the 3’-end.
An extended selection called X-selection crosses the results obtained in two designs (e.g. in two distinct databases) (see GUI next session).

f. Input and Output

(1) BLAST database

CADO4MI requires BLAST formatted nucleotide databases for BLAST step. These databases should contain all the sequences against which you want to assess the oligonucleotide specificity. You can use for example RefSeq or UniGene version compatible with your organism of interest (human versions can be downloaded from CADO4MI web site) or create your own databases.
In order to simplify the analysis, we recommend you to create databases using fasta files with simple headers. For example the “>” should be followed directly by the **accession number** and the **definition** of your sequence.

Ex:
>gi|10863904|ref|**NM_004239**.1| **Homo sapiens thyroid hormone receptor interactor 11 (TRIP11)**
changed into :
>**NM_004239 Homo sapiens thyroid hormone receptor interactor 11 (TRIP11)**

We provide a simple tool to do this; **DBFastER** (free Tcl/Tk application available at http://bips.u-strasbg.fr/CADO4MI).

Then you can run formatdb (BLAST package) to format your fasta file into a BLAST database (see formatdb man pages for further details). We propose to use the parsing option of formatdb (**-o T**) to create indexes of each sequence. This will allow CADO4MI to search in your BLAST database for sequences and/or definitions (if present in the header) using the fastacmd program (BLAST package).

Ex : « formatdb -i RefSeq.tfa -t "RefSeq r16" -p F **-o T** -n RefSeq_Hs » will create the five following files:

RefSeq_Hs.nhr
RefSeq_Hs.nin
RefSeq_Hs.nsd
RefSeq_Hs.nsi
RefSeq_Hs.nsq

(2) Input

CADO4MI requires sequence under fasta-formatted files and support single or multiple sequence(s) per file (Appendix FASTA Format). We also recommend you to provide fasta files with simple headers (only the accession number).

Ex :
```
>NM_001100
CCACCGCAGCGGACAGCGCCAAGTGAAGCCTCGCTTCCCCTCCGCGGCGACCAGGGCCCGAGCCGAGAGT
AGCAGTTGTAGCTACCCGCCCAGAAACTAGACACAATGTGCGACGAAGACGAGACCACCGCCCTCGTGTG
CGACAATGGCTCCGGCCTGGTGAAAGCCGGCTTCGCCGGGGATGACGCCC…
```

(3) Output

CADO4MI generates also output files for each sequence submitted:

Step or specific task	File extension	File Format
1- PolyA masking	.masked	FASTA
2- Similarity search	.blastn	BLAST
3- Sequence analysis	.log	Text
	.oligo	FASTA
4- Probe selection	.selection	FASTA
Temporary	.working	Text
-	.parameters	Tab separated
Global result	-	Tab separated
Cross-selection	.croisee	FASTA

The input sequence is stored after the masking step (".masked") and the ".blastn" file contains the BLAST results. The report of the analysis (T_m, GC, and specificity) is stored in a log file and filtered oligonucleotides are saved in the ".oligo" file (multiple fasta-formatted file). The selected oligonucleotide is stored in the ".selection". The ".working" is a temporary file indicating that the query is being processed. Finally, all parameters used for the design are in a single file ("Design.parameters") stored in the directory of results once for a set of query sequences.

The user can also create two additional files. The first one corresponds to the selection between two designs (i.e. X-Selection, ".croisee" file) and the second one is a result file (tab-delimited file) containing one or all result of a set of sequence. This file contains positions, $T_{m,}$ the best hit in database, number of detected sequences, the sequence and accession numbers for each best oligonucleotide selected.
All these files allow a complete overview of the design information to the user and let him manually refine and validate the problematic oligonucleotides.

4. Graphical User Interface (GUI)

In order to be readily accessible to many users, CADO4MI is available via a Graphical User Interface (GUI). The GUI allows an easy access to all parameters (i.e. selection of particular regions of design) and advanced design for problematic cases (e.g. splice variants, highly homologous sequences). An integrated presentation of the results (see below) provides overviews with distribution charts (T_ms, GC and average percent identity and number of sequence detected by BLAST), relative location along the query and detailed features for each oligonucleotide.

In order to fully exploit the GUI, it is recommended to use a 3-button mouse. The reason is that many functions will be accessible by using all 3 buttons and also with any combination of “Shift” or “Control” keys. These combinations provide access to a maximum of nine possibilities (see example below).

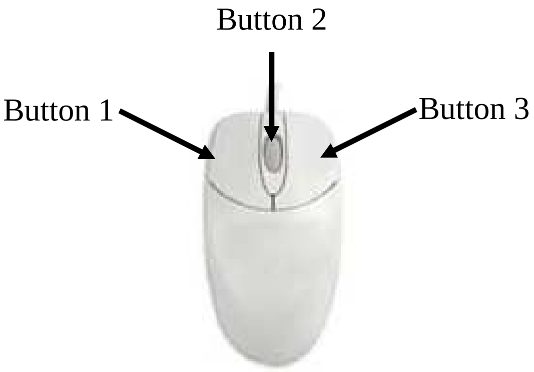

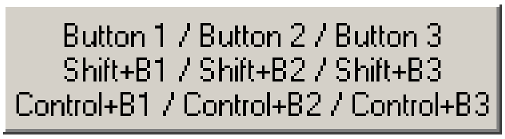

If only one action is available default is to use “Button 1”, if a second action is available it will be “Button 2” etc…

Help or complementary information is always available through “Help bubbles” by simply holding the mouse on a label or button.

a. Start Panel

The program starts with the main panel giving access to the different aspects of CADO4MI and to a log panel.

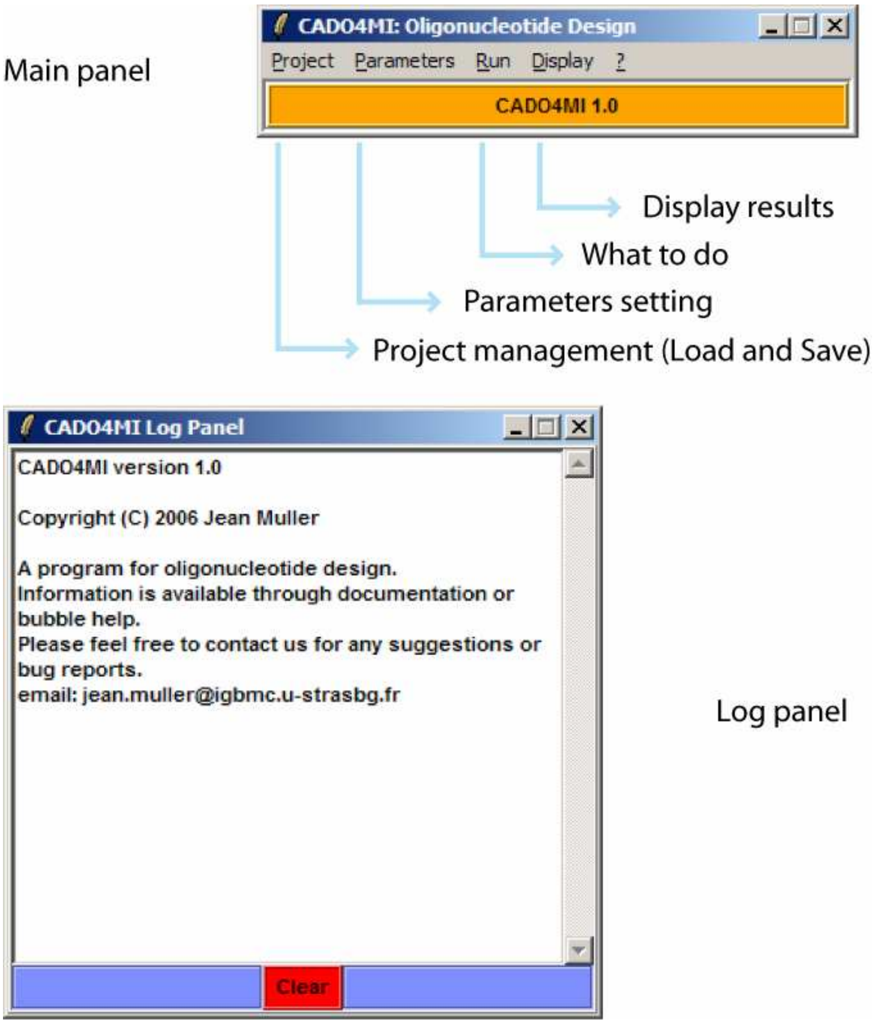

Using the menu "Parameters" you can display the settings to design oligonucleotides (Oligo Design Menu) and by using the menu "Display" you can access to the visualization of design results (Design Result Menu). These 2 main menus are the main entry points in CADO4MI.

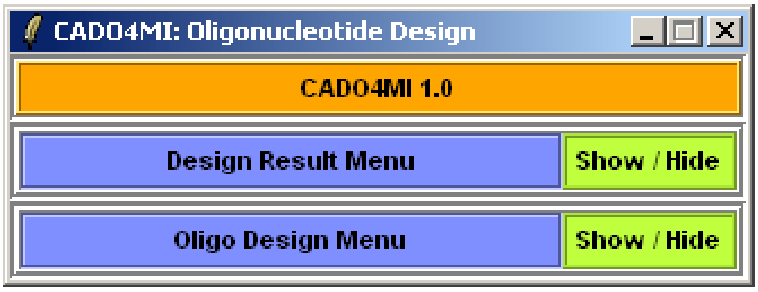

b. Oligo Design Menu

The Oligo Design Menu is divided into several parts:

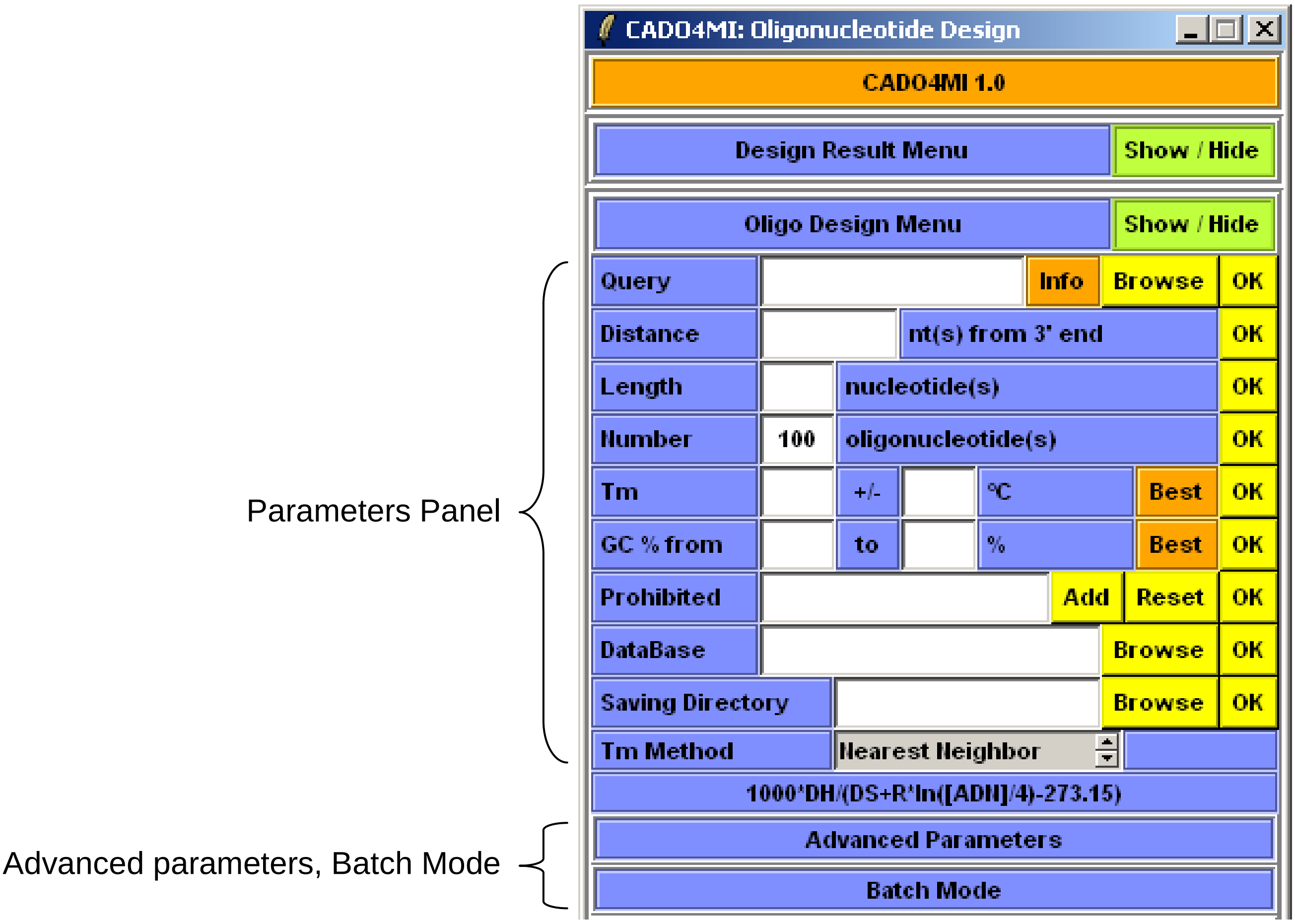

(1) Parameters Panel

The "Parameters Panel" is an easy access to all criteria important for the design of oligonucleotides. The user will be able to select his sequence(s) and adapt all criteria to his set of sequences.

- Query: Defines the set of query sequences. It must be in fasta-formatted sequence file(s) (Appendix Fasta Format). You can also use multiple fasta files (a specific Dialogue Box will allow you to separate them).
- Info: A graphical view of your sequence(s) with basic ATGC counts and distribution charts. This is also an easy way to choose precise areas of design (see appendix Information Panel).
- Distance: Defines the maximum distance from 3' end to use for the design. It is applied to all sequences of the user defined set.
- Length: Defines the size of the oligonucleotide to design. It corresponds also to the size of the sliding window used in any calculations.
- Number: Defines the number of oligonucleotide to keep at the end of the process. If you want to keep all, you would set it to 100000.
- T_m: Defines the T_m (melting temperature) range to use (e.g. 92±5 °C).

- GC: Defines the GC range to consider (e.g. 30 - 70 %).
- Best: Available for T_m and GC computation allows the user to plot all values computed for the set of sequences selected. The values are computed by moving a sliding window defined by the user (default size is “Length” and 10 for Step, see advanced parameters) and plotted as interactive graphs (See Appendix Parameter estimation).
- Prohibited: Defines sequences to avoid from the oligonucleotides (e.g. AAAAA...)
- Database: Defines the BLAST database to use for the specificity step. You may use nucleotide databases build with formatdb program (e.g. “.nsq”). If you want to redesign oligonucleotide and keeping the blastn files you should enter “none”.
- Tm method: Defines the method to calculate the T_m of your sequence.
- Saving directory: Defines the directory where to store the results.

(2) Advanced Parameters

In addition to the previous parameters, CADO4MI allow the user to fully customize all critical parameters such as Specificity or Step. This is achieved via the advanced parameters.

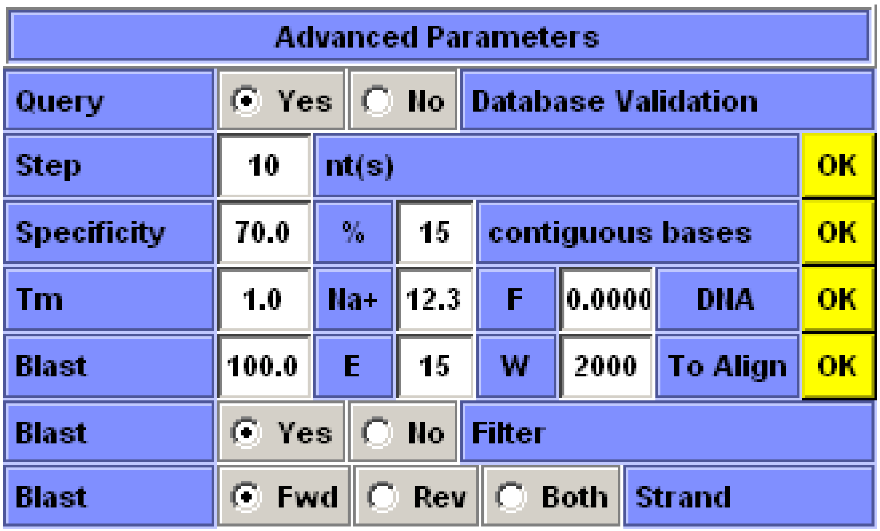

- Query: Defines if the query validation module is active or not. It is advised to use it in regular cases but for example if you decide to check your sequences against a genome this should be disabled.
- Step: Defines the step to move the sliding window (default 10 bases).
- Specificity: Defines the two specificity criteria (Kane’s rule) (defaults are 70% identity and 15 contiguous bases).
- Tm: Defines salt molar concentration (Na^+, default 1), formamide concentration (F in %; default 12.3%) and sequence molar concentration (DNA, default 0.000001) for T_m calculation.
- Blast: Defines maximum expect (E), size of the word (W) and maximum sequences to align (To align).
- Blast: BLAST Filter parameter (default is active).
- Blast: Defines which Strand to search in database (default forward only).

(3) Batch Panel

CADO4MI also includes a “Batch Mode” to run each step of the protocol separately. In addition, “X-Selection” performs automatic selection by crossing the results of two designs and “Save Results” permits to save all results from a design in a tab-delimited file.

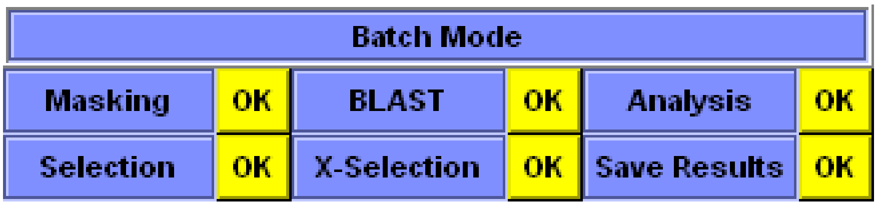

c. Design Result Menu

The results of the design can be visualized within the “Design Result Menu” by either selecting directly your result file (“File:” and “Browse button”) or by selecting the directory containing the design (“Directory:” and “Browse button”). This latter permit to show all sequences submitted and the selection of all or only a subset of them. You can also enter or select different directories and display for the same query the results of several designs. A combination of “File” and “Directory” entry will give priority to the name of the file but use directory to look for it.

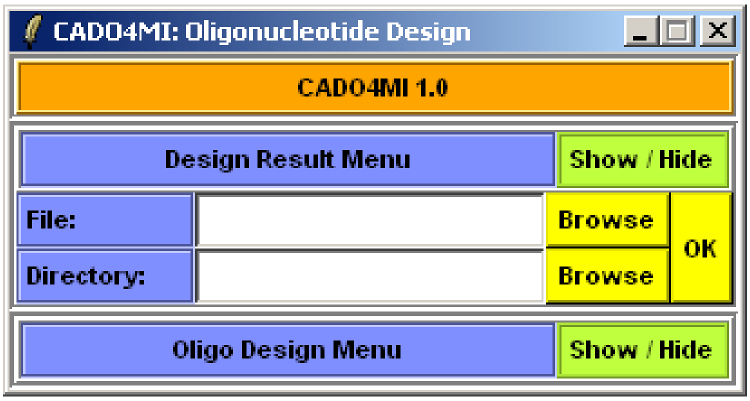

For each query, the results are shown within a window divided into three parts, an “Interactive board” 1 with the query (large red rectangle) and the profiles (T_m and GC content), the “Design board” 2 containing the results (many short and colored rectangles finished by the name of the directory) and the “Buttons board”. 3

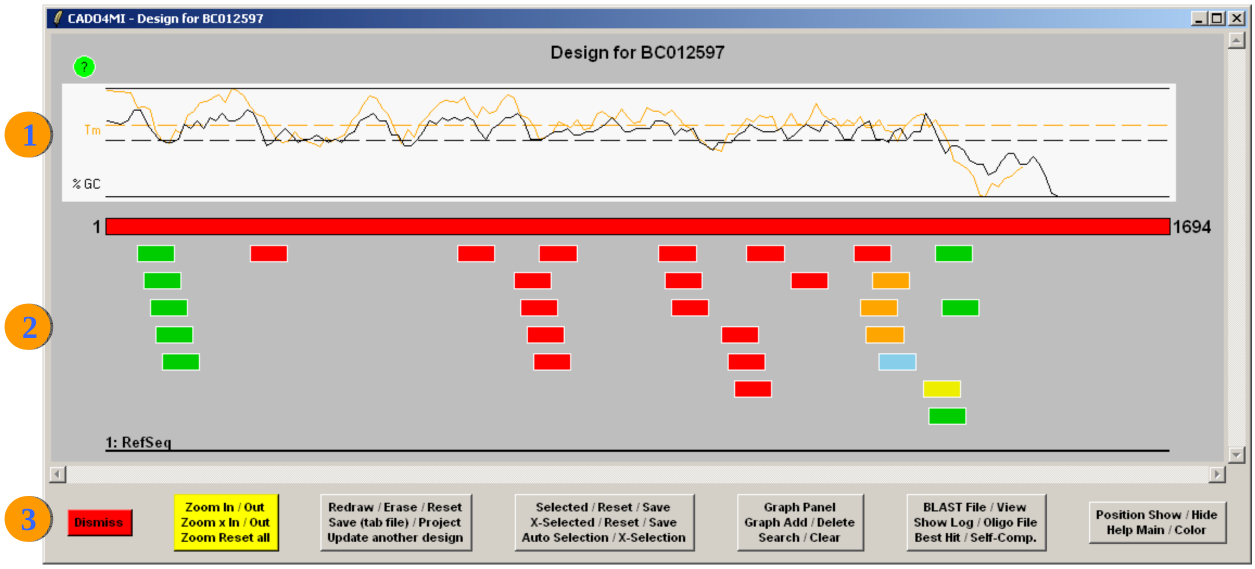

(1) Interactive board

The "Interactive board" works as the one describe for the "Information Panel" (Appendix Information Panel) with the profiles and the Flag for positions on the query. Values can be visualized by simply moving you mouse over it.

(2) Design board

The "Design board" represents oligonucleotides (short rectangles) localized according to their positions on the query and colored according to their number of detected sequences (see scheme below).

Number of potential target					
(white)	0	(green)	1	(light blue)	2
(yellow)	3-5	(orange)	6-8	(red)	>8

This panel is fully interactive and many functions and information are available for the different objects. We will consider **Oligonucleotide**, **Query** and the **board** it self as 3 objects. Some properties of these objects can be accessed using the 3 mouse buttons (see below).

Oligonucleotide:

By simply moving the mouse through the rectangle the user can display a short summary of oligonucleotide characteristics ("Info Bubbles").

	**Button 1**	**Button 2**	**Button 3**
	Positions	ID detected	Oligo sequence
**+ Shift**		Detailed ID detected	
**+Control**	Select the current oligo	X-select the current oligo	

Using “Button 2” or “Shift+Button 2” you can display respectively a simple list of the detected IDs by the corresponding oligonucleotide and a detailed view enhanced with the percent identity and the number of contiguous identical bases for each detected ID. Then, using the FastaCmd button, the user can retrieve the corresponding header and/or sequence. The header can be equivalent to the definition (if correctly formatted).

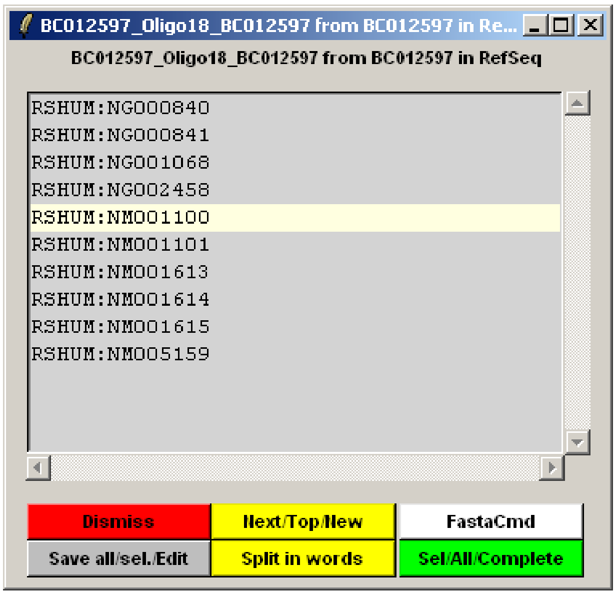

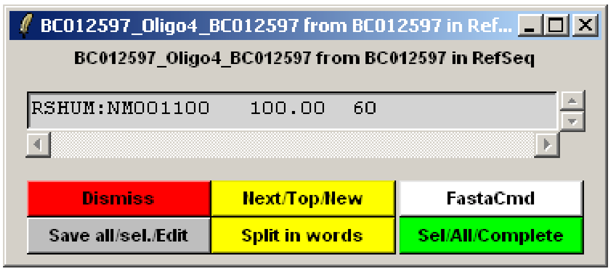

Ex: Definition retrieved for NM_001100 using the FastaCmd command.

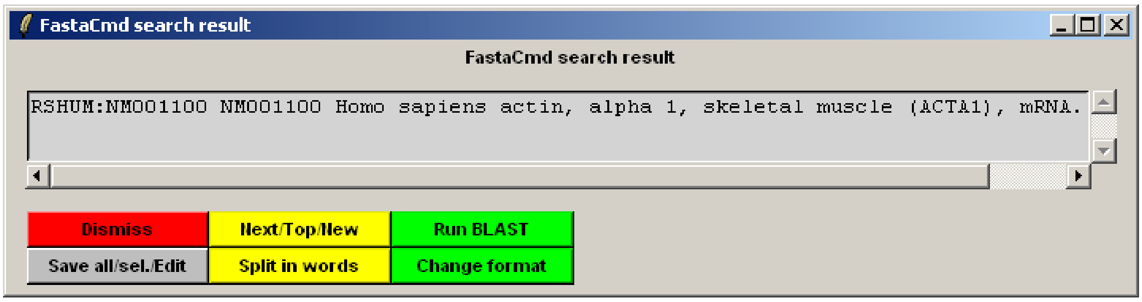

Ex: Complete sequence retrieved for NM_001100 using the FastaCmd command.

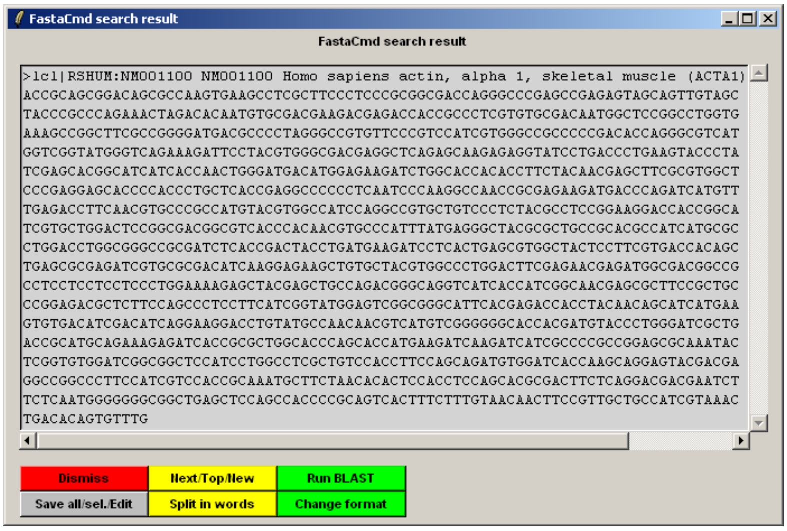

Query:

	Button 1	Button 2	Button 3
	General sequence information		Query sequence
**+ Shift**			
**+Control**			

Board:

	Button 1	Button 2	Button 3
			Hold display a zoom box
**+ Shift**	Display a cross line for localization (“Moving Flag”)		
**+Control**			

The “Design board” is also able to display several results for the same query at the same time. For example, the user can compare the results for the same sequence in two databases (here RefSeq and UniGene):

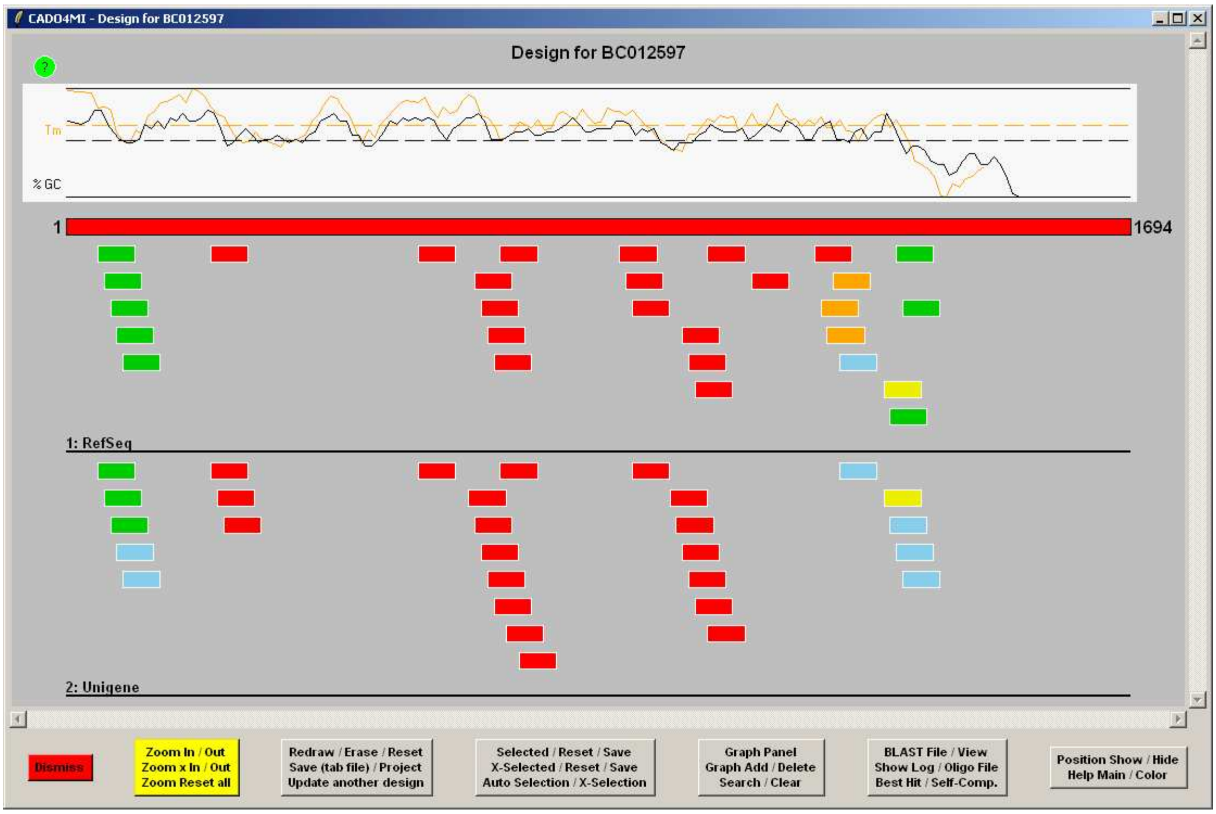

To select multiple directories you can use several times the “Browse button” (the browsing results are concatenated) or enter two (or more) directories in the “Design Result Menu” (Copy paste is also working).

Example:

This example shows many interactive features:

- Selected oligonucleotide (black discontinuous outline)
- X-Selected oligonucleotide (black cross)
- Oligonucleotide found by text search (violet discontinuous outline)
- Positions of oligonucleotides
- Display of important criteria for one oligonucleotide (ivory “Info Bubble”)
- The Moving Flag

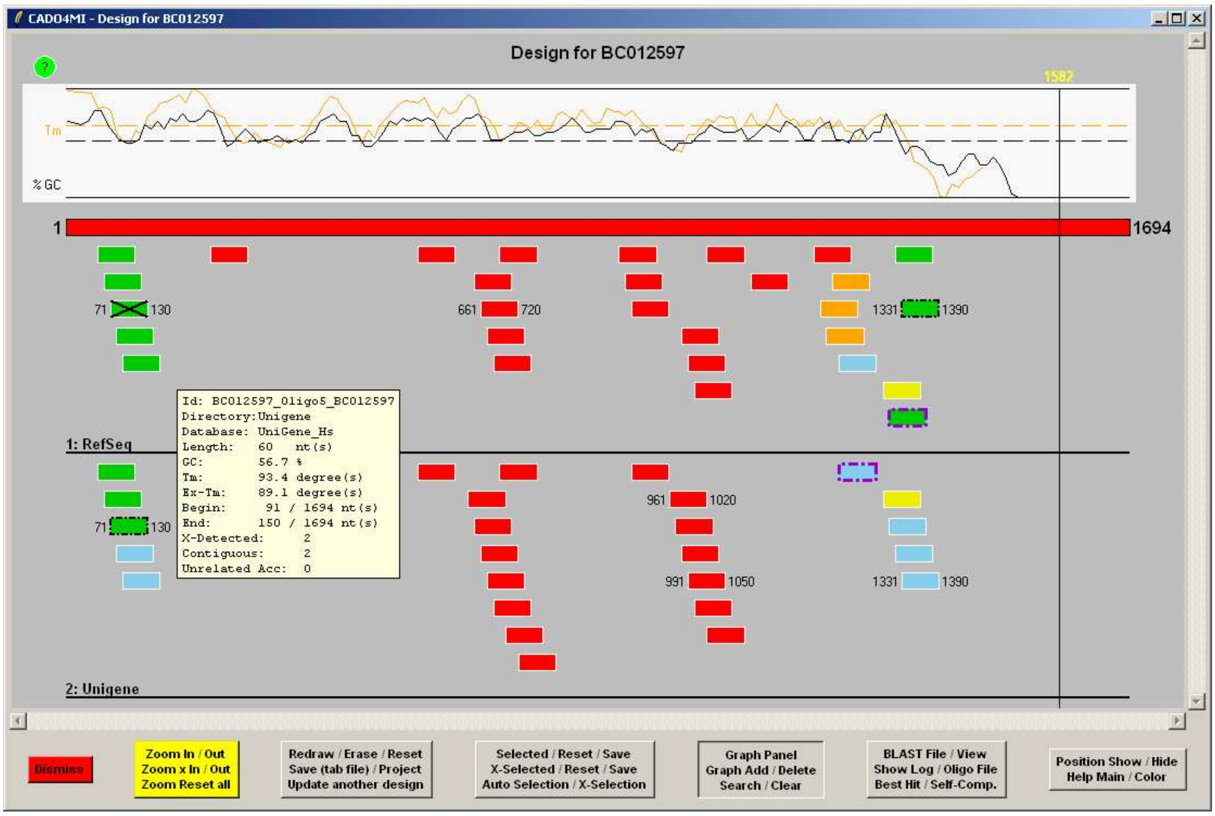

Information Bubble (“Info Bubble”) for one oligonucleotide:

```
Id: BC012597_Oligo5_BC012597
Directory:Unigene
Database: UniGene_Hs
Length:     60    nt(s)
GC:         56.7 %
Tm:         93.4 degree(s)
Ex-Tm:      89.1 degree(s)
Begin:       91 / 1694 nt(s)
End:        150 / 1694 nt(s)
X-Detected:       2
Contiguous:       2
Unrelated Acc:    0
```

Detailed information; oligonucleotide identifier, directory where the design is saved, database (blast) used during the design, oligonucleotide length, GC content and T_m values (“T_m“ is the one selected for the design and “Ex-T_m“ is comparative value calculated using the DNADNA method), positions along the query, number of sequence detected (Target sequence + non-target sequence(s)), number of detected sequences due to only Contiguous parameter.

(3) Buttons Board

The “Button board” integrates a lot of functions which are described with all possibilities.

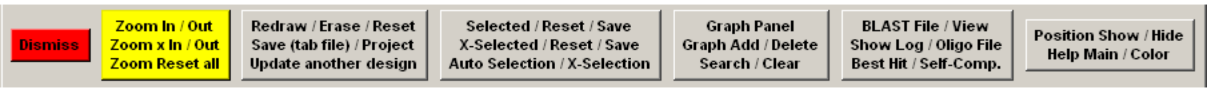

-Display:

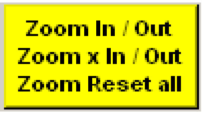

	Button 1	Button 2	Button 3
	Zoom in	Zoom out	
**+ Shift**	Zoom in (X axis)	Zoom out (X axis)	
**+Control**	Reset zoom to initial position		

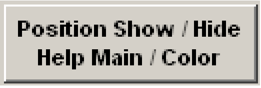

	Button 1	Button 2	Button 3
	Display oligo positions	Hide oligo positions	
**+ Shift**	Display a short help	Display color scheme	
**+Control**			

- Graph and others:

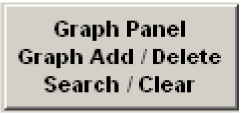

	Button 1	Button 2	Button 3
	Display Graph Panel		
**+ Shift**	Display add Graphs	Display delete Graphs	
**+Control**	Search oligo by text	Clear search selection	

For “Graph Panel” see also the appendix corresponding section.

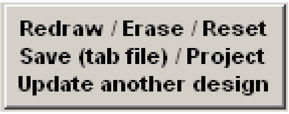

	Button 1	Button 2	Button 3
	Redraw the results	Clear display	Clear results from memory.
**+ Shift**	Save current results in a tab-delimited file	Save project results	
**+Control**	Update another design with selection of the current design		

The two "save" function use the same mechanisms as the one present in "Batch Mode".

<u>-Selection:</u>

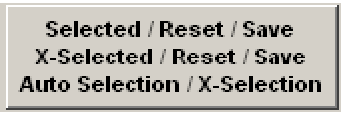

	Button 1	Button 2	Button 3
	Display selected oligo	Clear selection	Save selection
**+ Shift**	Display X-selected oligo	Clear X-selection	Save X-selection
**+Control**	Compute selection	Compute X-selection	

Note: you can manually select your desired oligonucleotide see above (Control+1 or Control+2 directly on the oligonucleotide).

<u>-Blast analysis and others:</u>

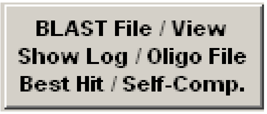

	Button 1	Button 2	Button 3
	Display raw blast	Graphical blast view	
**+ Shift**	Display Log file	Display Oligo file	
**+Control**	Best Hit in database	Self Complementary	

"Best Hit in database" is the sequence validation function to check if the query sequence is present or partially present in the database. This is done automatically during the design process but can be recomputed or simply rechecked here (Appendix Sequence validation).
"Self Complementary" is a simple and fast algorithm to check if the selected oligonucleotides have self complementary sequences.

5. Command line

CADO4MI is also available via command lines in order to be accessible into other scripts, see the list below:

-q (Query)	Your query sequence in fasta format [String]
-l (Length)	Length of designed oligonucleotide (and size of sliding window) [Integer]
-de (Distance)	Distance from 3' end of all queries for restricted designing area(s) [Integer]<br>ex: 2000 restricts the design between End-2000 to the End of your query
-ar (Area)	List of begin-end points in all queries for restricted designing area(s) [Integer]<br>ex: 1 300 500 800 will allow design only within the two ranges
-nb (nboligo)	Number of oligonucleotide to keep at the end of process (default 100) [Integer]
-t (Tm)	Tm threshold (ex: 92.2) [Real]
-r (TmRange)	Tm range (ex: 5) [Real]
-m (TmMethod)	Tm calculation method [String]<br>NearestNeighbor (default)<br>Wallace<br>DNADNA<br>DNARNA<br>RNARNA
-gl (GCLower)	The minimum %GC accepted (ex: 35.0) [Real]
-gu (GCUpper)	The maximum %GC accepted (ex: 70.0) [Real]
-p (Prohib)	Prohibited sequence, must be a list of sequence (ex: AAAA TTTTT) [String]
-b (Database)	Blast Database to assess specificity (ex: .nsq) [String]
-w (WorkingDir)	Directory where to save your results [String]
-qc (QueryCheck)	Query validation in database with %Id and %Cover (1 or 0) [Integer]<br>Default is 1 (for yes).
-st (Step)	Value between each move of the sliding window (default is 10) [Integer]
-sp (Specificity)	Specificity threshold to prevent X-Hybridization [Real]<br>Please enter value as 70.0 (default value).
-nc (Contiguous)	Specificity threshold for max contiguous identical bases allowed [Integer]<br>Default is 15 contiguous bases rejected.
-ts (Salt)	Salt concentration (default 1.0, see Tm) [Real]
-tf (Formamide)	Formamide (default 12.3 %, see Tm) [Real]
-tc (DNA)	DNA concentration (default 0.000001, see Tm) [Real]
-ev (BlastExpect)	The Blast E-value limit (default is 100) [Real]
-bw (BlastWord)	The word length for Blast searches (default is 15) [Integer]
-ba (BlastNbAligned)	The Blast sequence limit to align (default is 2000) [Integer]
-bs (BlastStrand)	Blast option forward (1, default), reverse (2) or both (3) strands [Integer]
-bf (BlastFilter)	Blast filter activation, T for yes (default), F for no [String]

Obligatory parameters are: Query, Length, Tm, Tm range, Database and your working directory.

Type "**CADO4MI –check**" for this help.

6. Appendix

FASTA FORMAT

A sequence in FASTA format begins with a single-line description, followed by lines of sequence data. The description line starts with a greater than symbol (">"). The word following the greater than symbol (">") immediately is the "ID" (name) of the sequence, the rest of the line is the description. The "ID" and the description are optional. All lines of text should be shorter than 80 characters. The sequence ends if there is another greater than symbol (">") symbol at the beginning of a line and another sequence begins.

The following example contains two sequences (Example1, Example2):

```
>Example1 envelope protein
ELRLRYCAPAGFALLKCNDADYDGFKTNCSNVSVVHCTNLMNTTVTTGLLLNGSYSENRT
QIWQKHRTSNDSALILLNKHYNLTVTCKRPGNKTVLPVTIMAGLVFHSQKYNLRLRQAWC
HFPSNWKGAWKEVKEEIVNLPKERYRGTNDPKRIFFQRQWGDPETANLWFNCHGEFFYCK
MDWFLNYLNNLTVDADHNECKNTSGTKSGNKRAPGPCVQRTYVACHIRSVIIWLETISKK
TYAPPREGHLECTSTVTGMTVELNYIPKNRTNVTLSPQIESIWAAELDRYKLVEITPIGF
APTEVRRYTGGHERQKRVPFVXXXXXXXXXXXXXXXXXXXXXXVQSQHLLAGILQQQKNL
LAAVEAQQQMLKLTIWGVK

>Example2 synthetic peptide
HITREPLKHIPKERYRGTNDTLSPQIESIWAAELDRYKLVKTNCSNVS
```

Sequences are expected to be represented in the standard IUB/IUPAC amino acid and nucleic acid codes, with these exceptions: lower-case letters are accepted and are mapped into upper-case; a single hyphen or dash can be used to represent a gap of indeterminate length; and in amino acid sequences, U and * are acceptable letters (see below). Before submitting a request, any numerical digits in the query sequence should either be removed or replaced by appropriate letter codes (e.g., N for unknown nucleic acid residue or X for unknown amino acid residue).

The nucleic acid codes supported are:

A	adenosine	M	A C (amino)
C	cytidine	S	G C (strong)
G	guanine	W	A T (weak)
T	thymidine	B	G T C
U	uridine	D	G A T
R G A	(purine)	H	A C T
Y T C	(pyrimidine)	V	G C A
K G T	(keto)	N	A G C T (any)

- gap of indeterminate length

SEQUENCE VALIDATION.

According to the heterogeneous origin of query sequences, we add a sequence validation module that will warn the user if the design result is reliable or not.
The query sequence is compared to the best hits found in the BLAST searches. The best hits are defined within the sequences with the highest number of nucleotide aligned. For each of these sequences a global percent identity (GID) and two percent coverage (pCover) are calculated. A warning is displayed if no detected sequences have >95% identity and >70% sequence coverage with the query. For calculation details see Appendix BLAST:

CADO4MI warns the user of such cases during results visualization and a report is available in the ".log file" and also if the user generates a result file.

Examples:

1. Here no sequences fulfill the criteria and so the design can be problematic and should be further validated by the user.

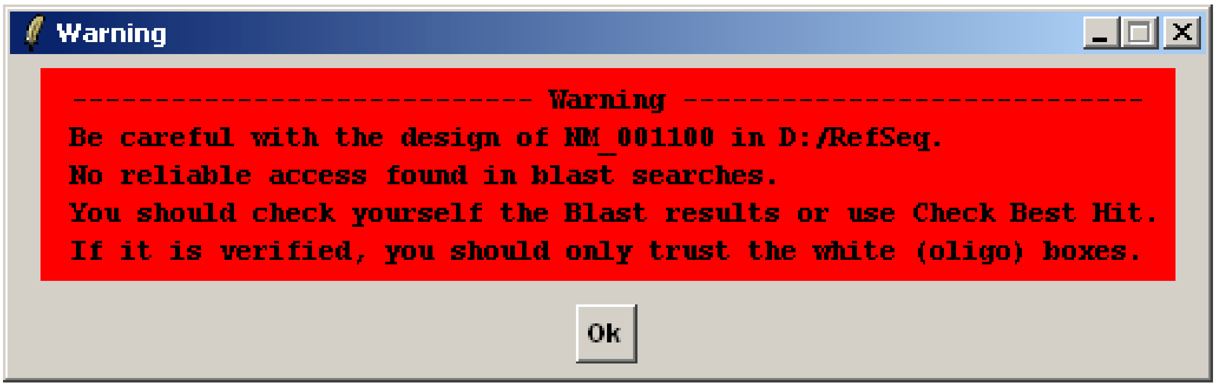

2. Here one sequence is identified as the equivalent sequence to the query.

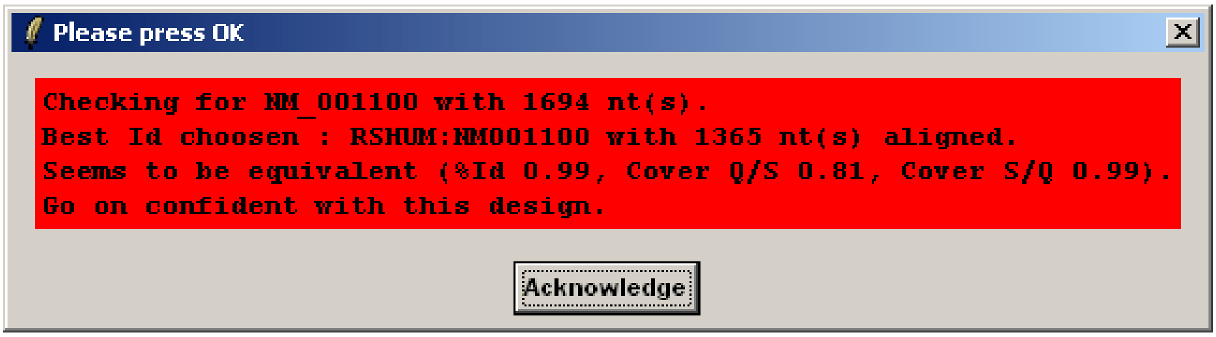

INFORMATION PANEL

The “Information Panel” is an enhanced graphical view of your sequence and permits to display profiles (T_m and GC) and to choose easily dedicated areas of design. Basic nucleotides counts (ATGC count ...) and T_m calculations (here using the complete sequence) are also available.
To show this panel for your query(s), push the “Info button”

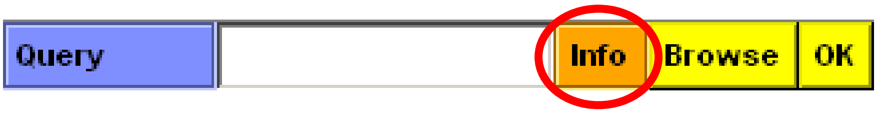

The query sequence is drawn as a red rectangle, the upper region contains the GC and T_m profile and represents the "Interactive board" (Appendix Graph Panel).

Interactive board

Query

Basic counts

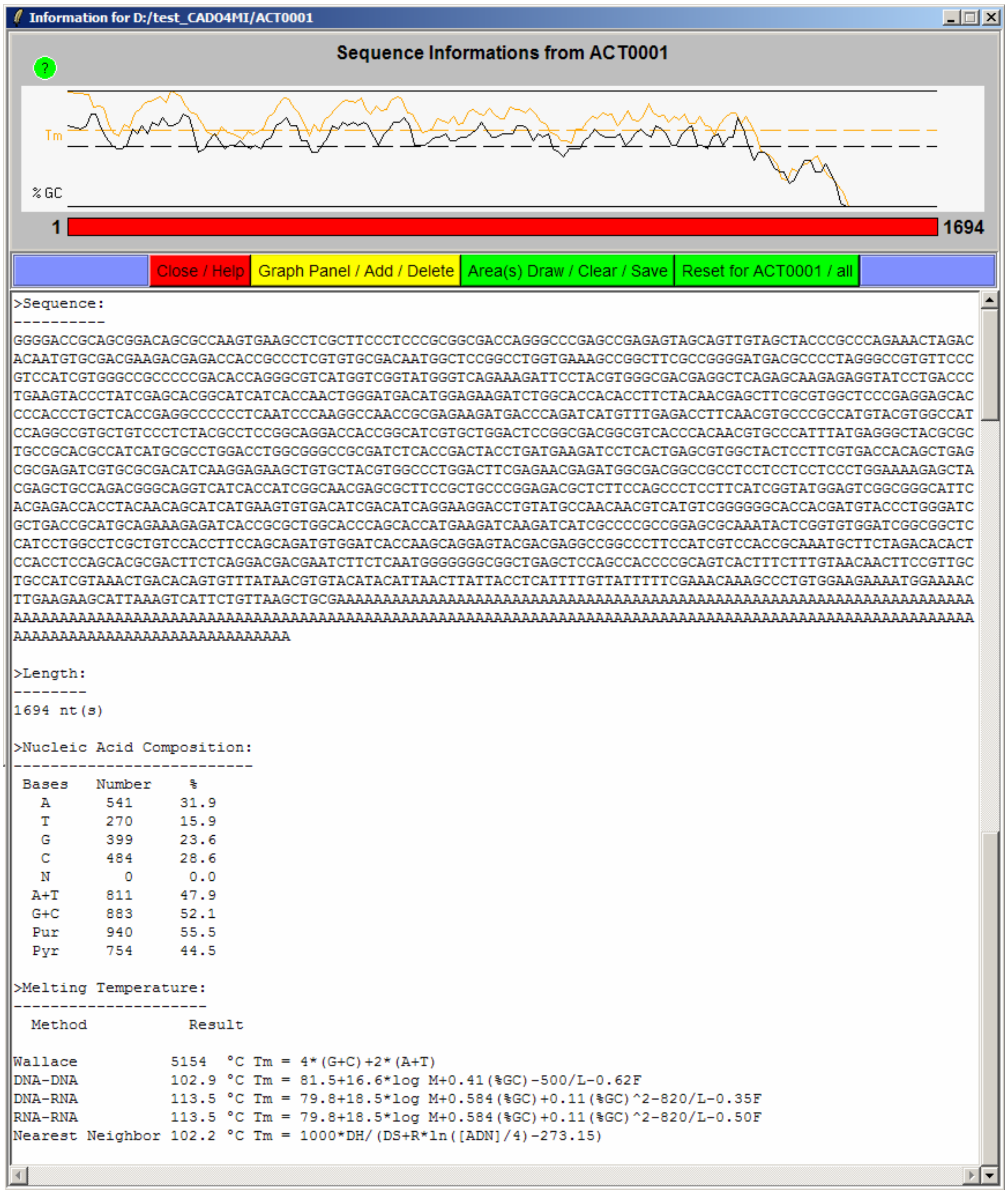

Area Selection:

Area selection is achieved by clicking on "Control+Button 1" on the "Interactive board" which display a flag (“Selection flag”) at the given position. One area is defined by two “Selection flags”. The flag can be erased by clicking again on "Control+Button 1" at the same position. Relative position of the flag is displayed by simply moving the mouse on it.

Another flag called “Moving flag” can be displayed by holding "Shift+Button 1" on the “Interactive board” and can be moved along the query. The “Moving flag” combined with “Selection flag” is designed to help the user to localize precisely positions of design.

The following buttons are used to save, redraw, or erase a selection.

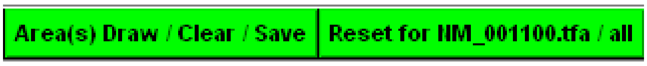

Draw: draw previously selected and saved areas.
Clear: erase drawn areas without cleaning the memory.
Save: save in memory your selection for the current sequence.
Reset: erase from the memory the previously saved areas for the current sequence.
All: same as Reset but for all submitted areas in all queries.

Examples:

1. In black, the “Moving flag” is displayed with relative position to the query in yellow.

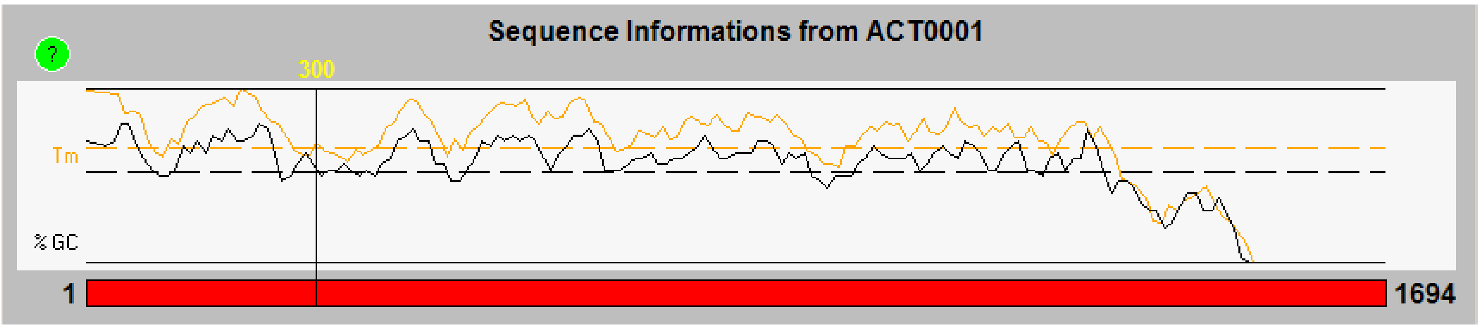

2. In green, four “Selection flags” are displayed (and can be saved). The position of the flags can be displayed using the mouse (see the 4th flag).

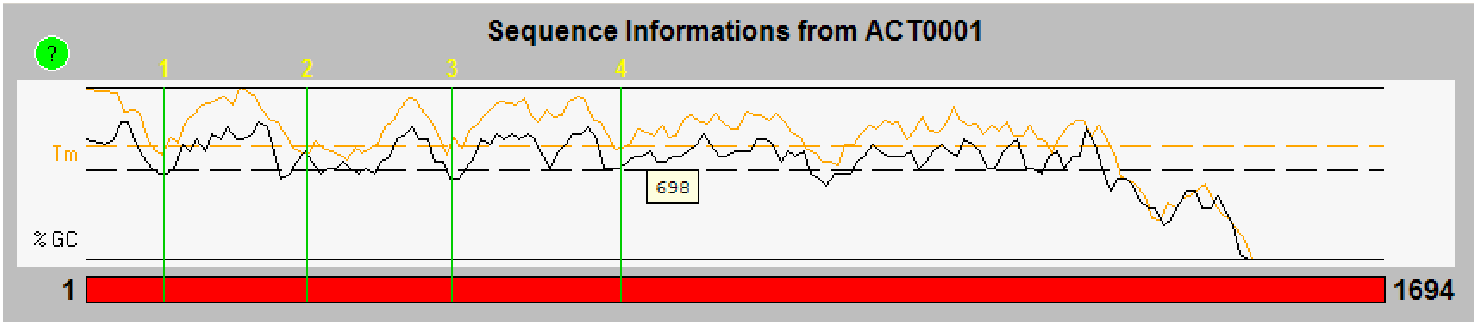

3. In blue the same four flags reloaded with the Draw Button:

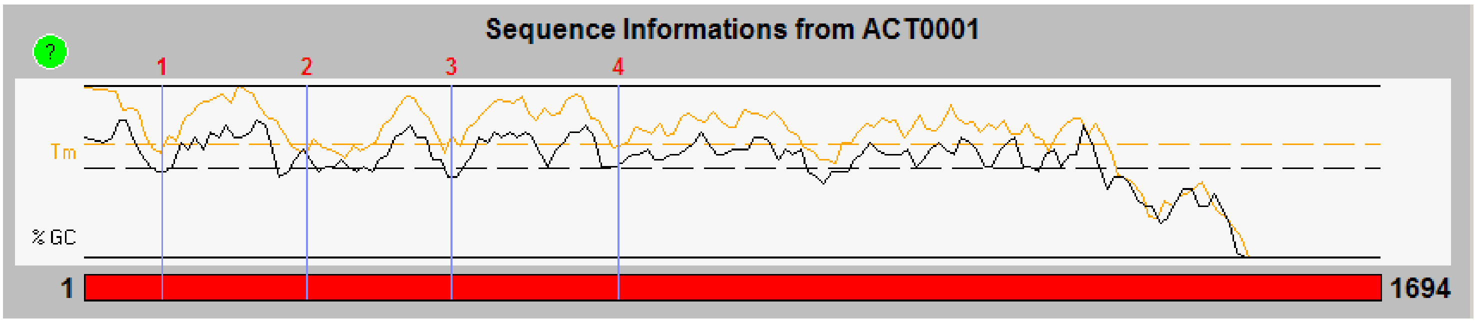

GRAPH PANEL

The Graph Panel is an interface that allows the user to configure the distribution charts ("graph") display (color, presence).

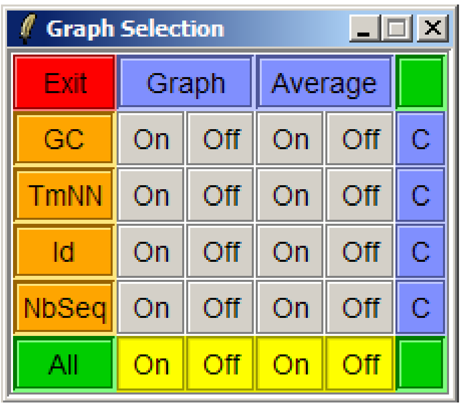

The names of the graphs are displayed on the left part of the window, and you can choose to hide or show one or all graph (Graph On/Off), one or all average (Average On/Off). You can also change their colors (C).

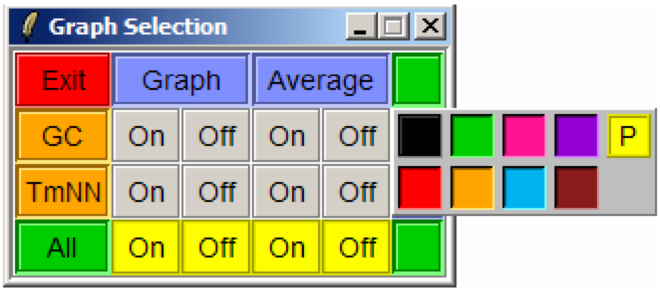

The Graph Button permits also to add or delete graphs. You can calculate and display five graphs for T_m (one for each method), one for the GC content, and two dedicated to Blast analysis (mean percent identity and the number of sequence aligned).

Examples:

1. This menu is used to add or delete graphs:

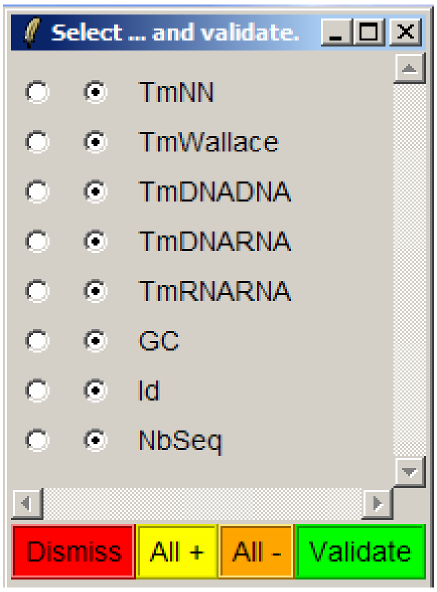

2. Here you can see two new graphs added; in blue, the number of sequence detected in database and in red, the mean percent identity of detected sequences.

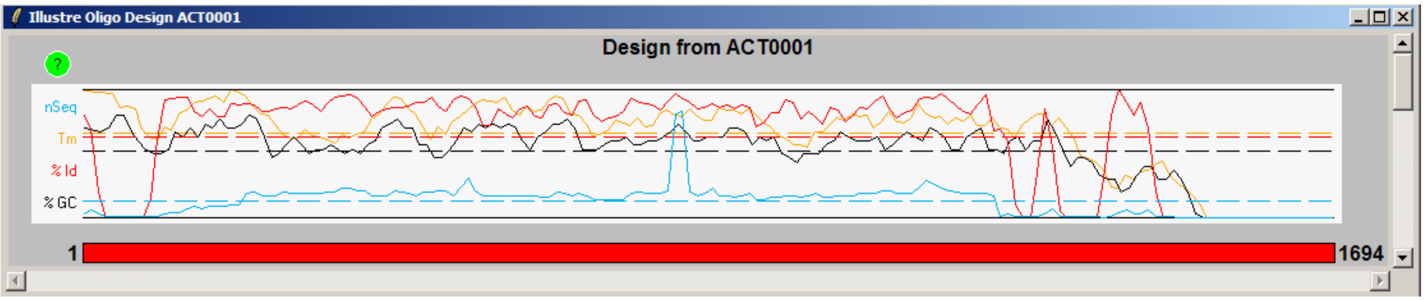

Note: In order to show interesting areas for possible specific oligonucleotide, the percent identity is set to 0 when the sole sequence detected is the query sequences.

PARAMETER ESTIMATION

Estimation of parameters is important to optimize results (Li and Stormo, 2001), CADO4MI offer the possibility to plot an interactive graph including all computed T_m and/or GC values (user defined sliding window) for a subset of or all sequences selected.
For example, the best T_m range (or GC) can be defined as the range of T_m belonging to the maximum number of sequences and can be directly chosen by the user via the GUI.

The interactive graph represent the values (x axis) and their count (y axis). The red line represents the mean. The user can also define Flags (Appendix Info Panel) and save them. These results can also be saved under file (tab-delimited file) to be reused in other programs (e.g. spreadsheet programs) to reproduce the graph and/or compare different graphs.

Examples:

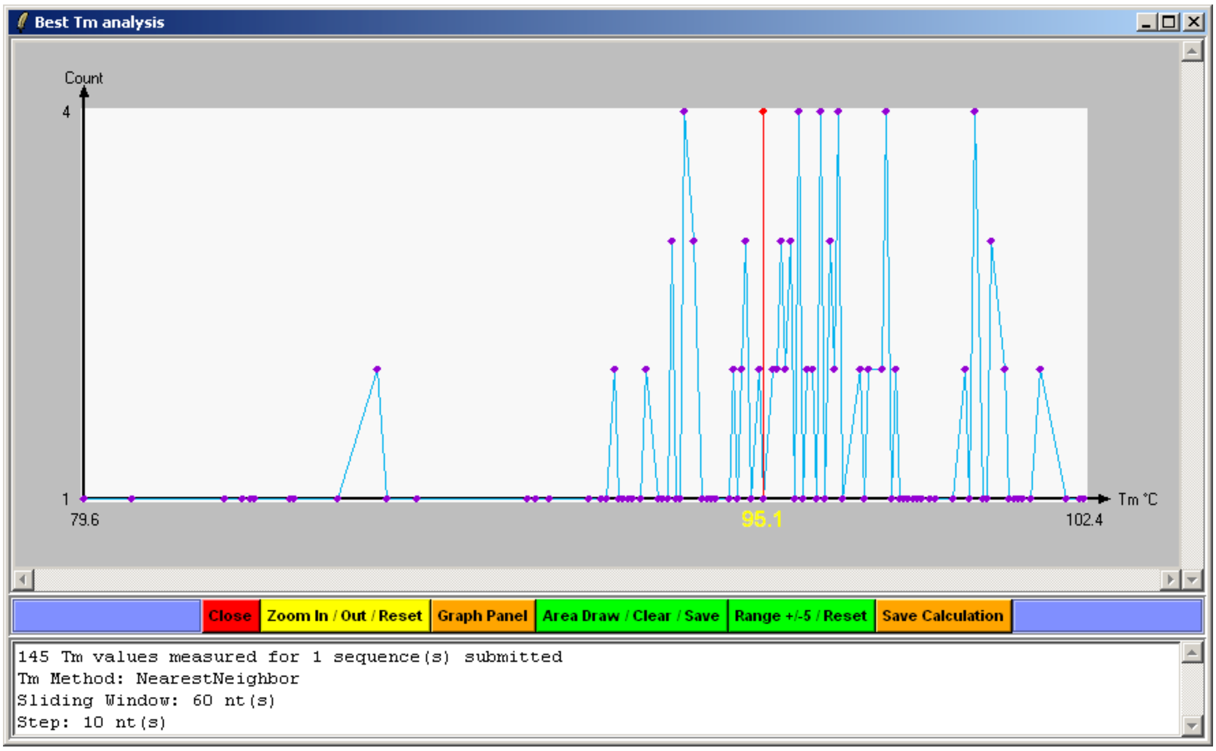

1. T_m calculation with Nearest Neighbor method for a single sequence.

2. T_m calculation with the Nearest Neighbor method for all sequences in RefSeq database release 1.

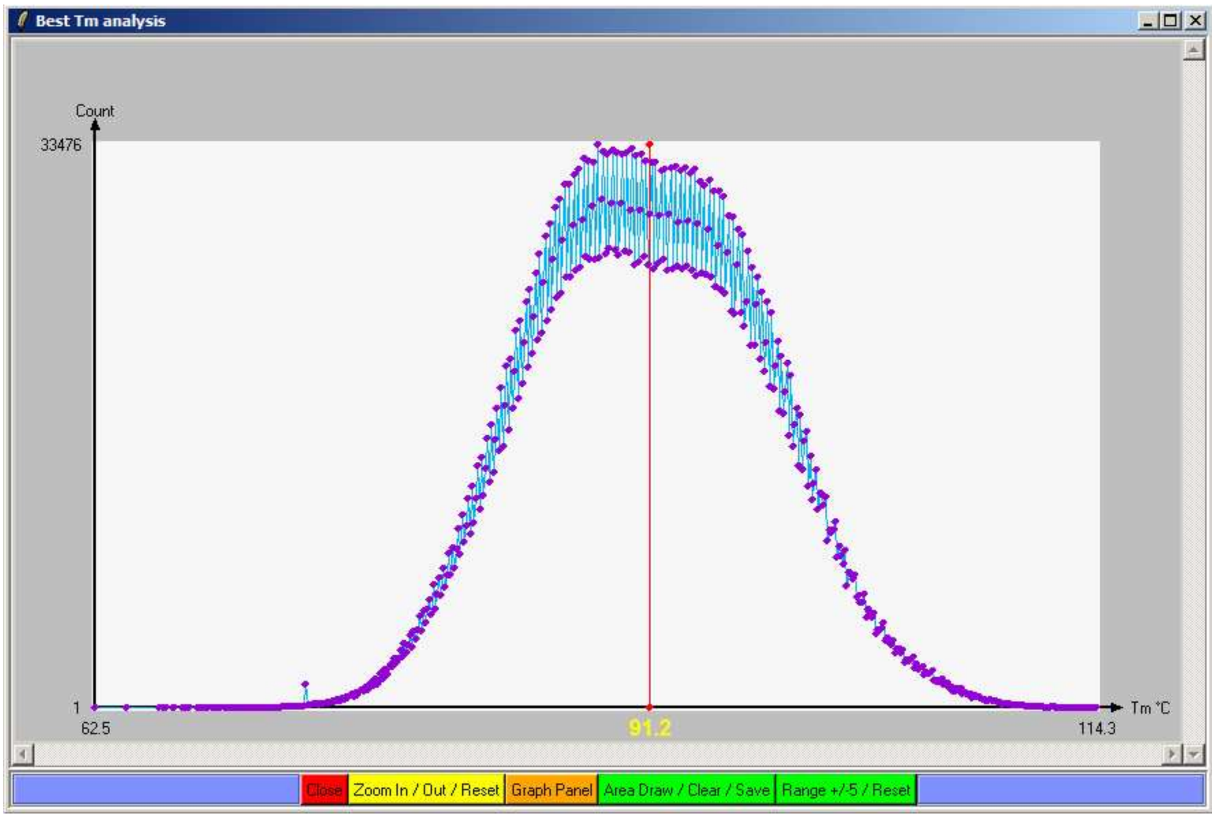

3. GC content calculation for a single sequence.

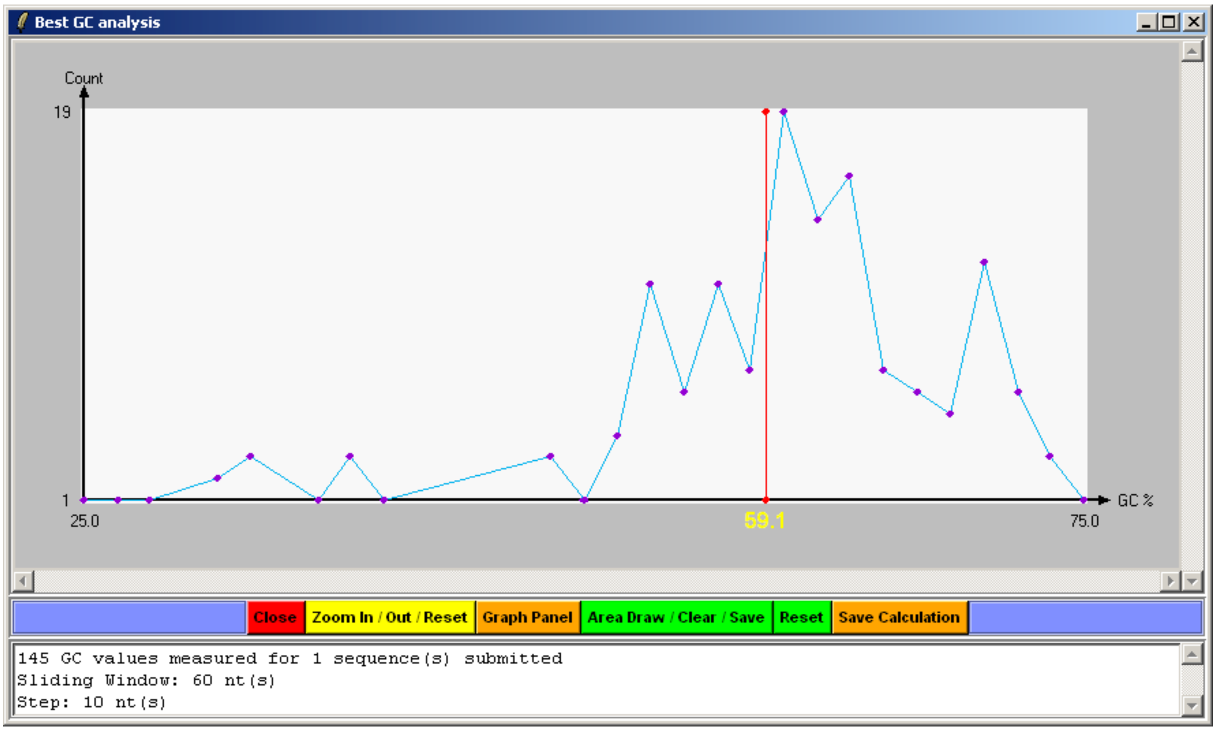

4. GC content calculation for all sequences in RefSeq database release 1.

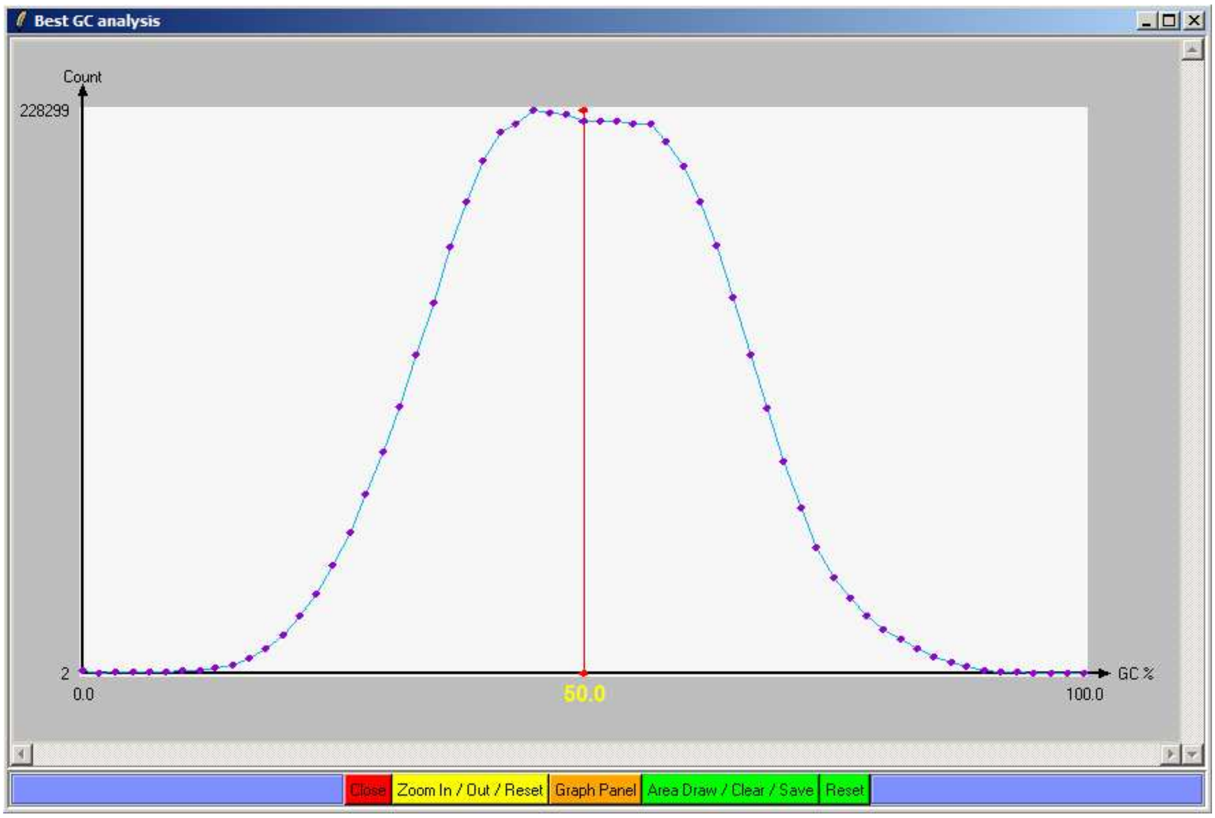

BLAST TOOLS

CADO4MI includes a dedicated BLAST parser (Chalmel et al, unpublished data) which allows treatment and visualization of BLAST results.

1. Graphical Interface:

The interface is a two panel window containing blast "subjects" (the upper panel) in the same order as the raw file and HSP ("High Scoring Pair") drawn with positions relative to the query sequence. The second panel displays alignment coverage in the two ways (Query to Subject and vice versa) that can be updated for each subject by clicking on the Accession number with "Shift+Button 1".

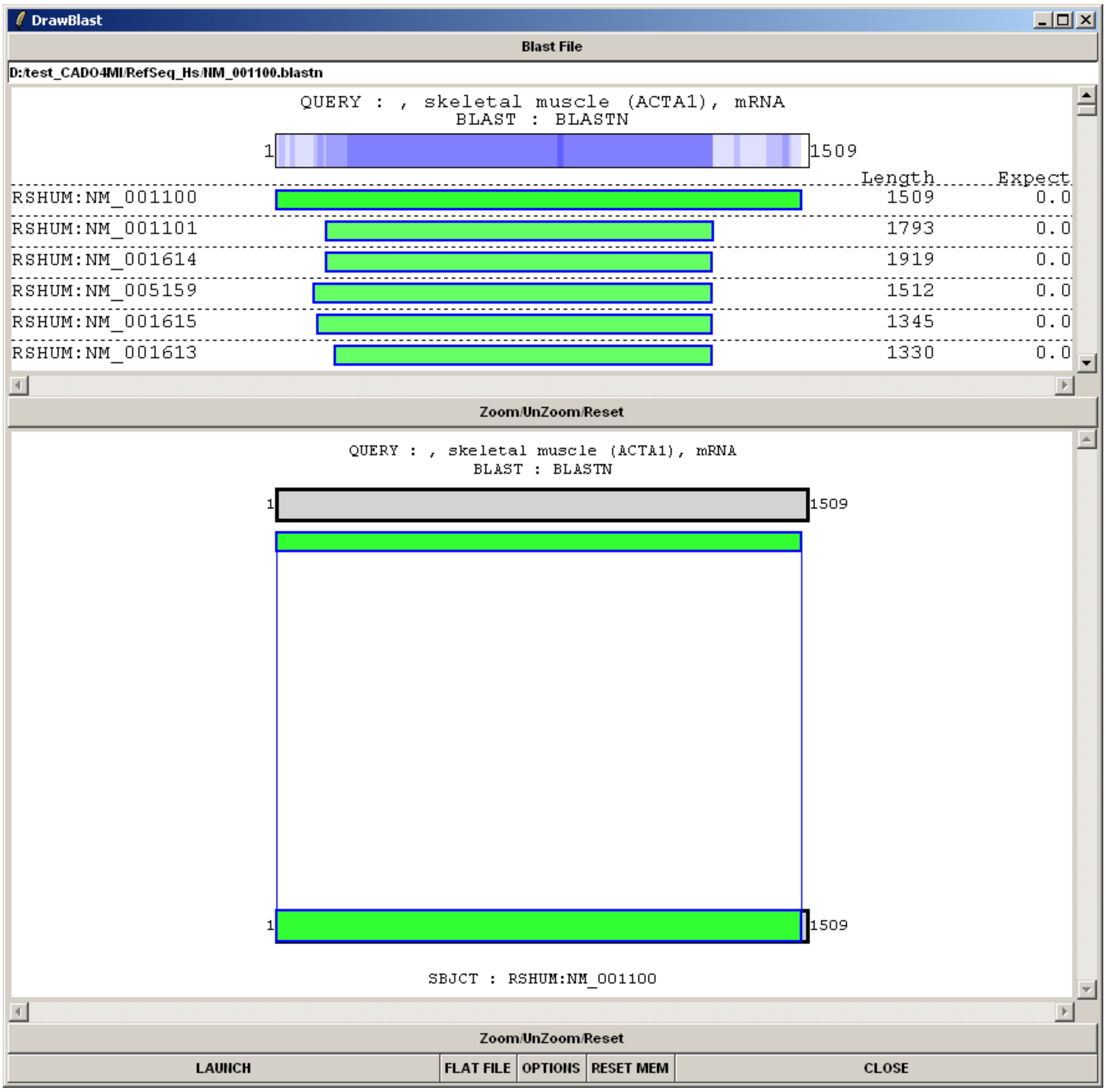

2. Percent Identity and percent coverage calculations:

The values described below are used to determine the best sequence within the blast output (See Appendix Sequence Validation).

<u>Global Percent Identity (GID):</u>

The global percent identity (GID) is the ratio of number of identical bases aligned to the maximum number of bases aligned.

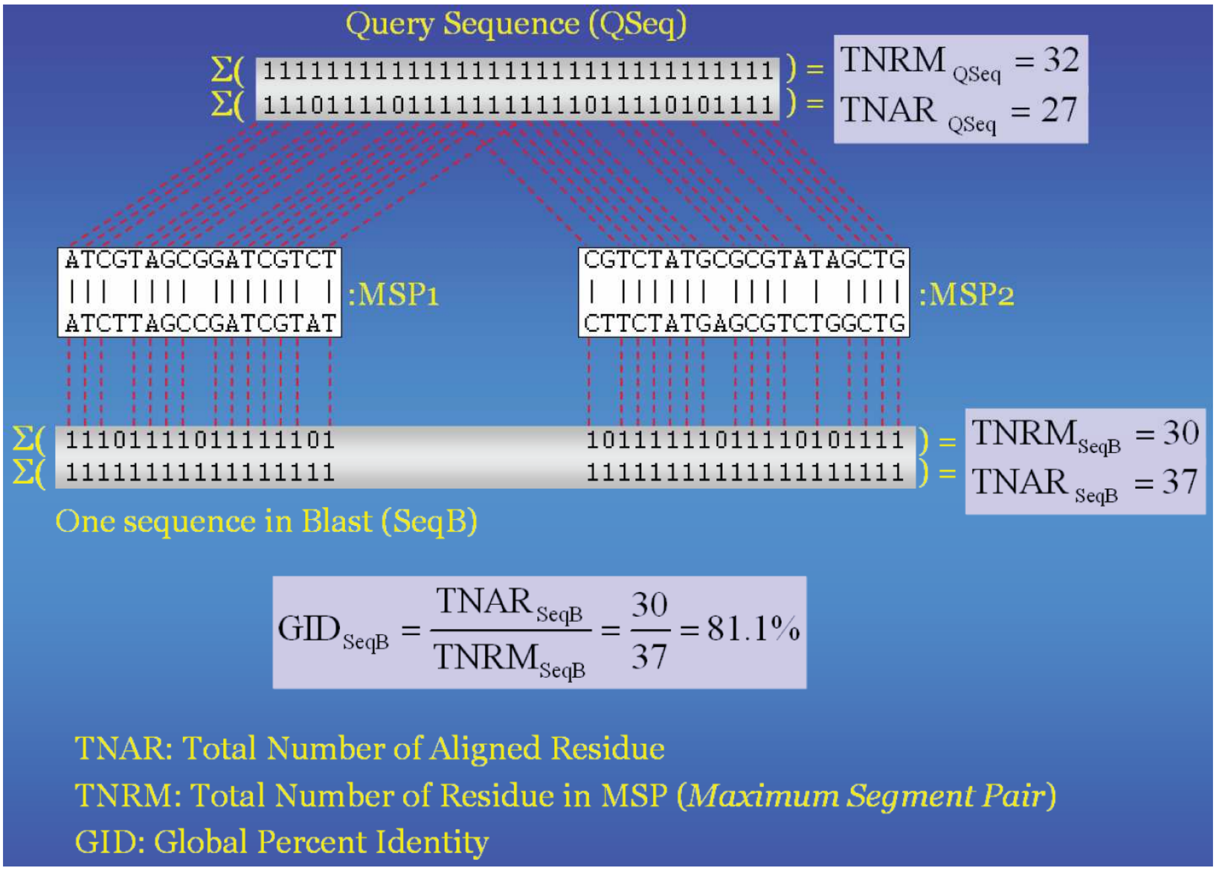

Percent coverage (pCover):

The percent coverage (pCover) is the ratio of number of bases aligned to the maximum number of bases that could have been aligned.

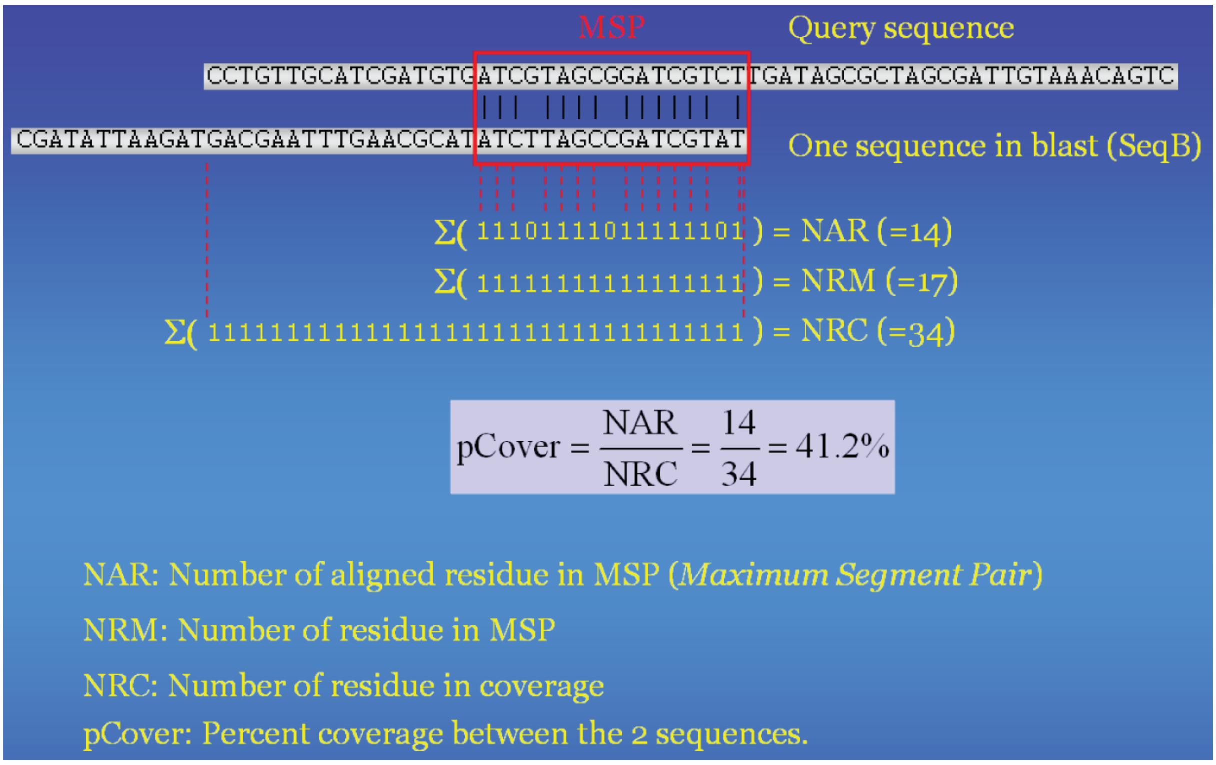

7. References

Altschul, S.F., et al. (1997) Gapped BLAST and PSI-BLAST: a new generation of protein database search programs, *Nucleic Acids Res*, **25**, 3389-3402.

Bodkin, D.K. and Knudson, D.L. (1985) Assessment of sequence relatedness of double-stranded RNA genes by RNA-RNA blot hybridization, *J Virol Methods*, **10**, 45-52.

Bolton, E.T. and Mc, C.B. (1962) A general method for the isolation of RNA complementary to DNA, *Proc Natl Acad Sci U S A*, **48**, 1390-1397.

Breslauer, K.J., et al. (1986) Predicting DNA duplex stability from the base sequence, *Proc Natl Acad Sci U S A*, **83**, 3746-3750.

Casey, J. and Davidson, N. (1977) Rates of formation and thermal stabilities of RNA:DNA and DNA:DNA duplexes at high concentrations of formamide, *Nucleic Acids Res*, **4**, 1539-1552.

Howley, P.M., et al. (1979) A rapid method for detecting and mapping homology between heterologous DNAs. Evaluation of polyomavirus genomes, *J Biol Chem*, **254**, 4876-4883.

Hughes, T.R., et al. (2001) Expression profiling using microarrays fabricated by an ink-jet oligonucleotide synthesizer, *Nat Biotechnol*, **19**, 342-347.

Kane, M.D., et al. (2000) Assessment of the sensitivity and specificity of oligonucleotide (50mer) microarrays, *Nucleic Acids Res*, **28**, 4552-4557.

Li, F. and Stormo, G.D. (2001) Selection of optimal DNA oligos for gene expression arrays, *Bioinformatics*, **17**, 1067-1076.

Meinkoth, J. and Wahl, G. (1984) Hybridization of nucleic acids immobilized on solid supports, *Anal Biochem*, **138**, 267-284.

Rychlik, W., et al. (1990) Optimization of the annealing temperature for DNA amplification in vitro, *Nucleic Acids Res*, **18**, 6409-6412.

SantaLucia, J., Jr. (1998) A unified view of polymer, dumbbell, and oligonucleotide DNA nearest-neighbor thermodynamics, *Proc Natl Acad Sci U S A*, **95**, 1460-1465.

Wallace, R.B., et al. (1979) Hybridization of synthetic oligodeoxyribonucleotides to phi chi 174 DNA: the effect of single base pair mismatch, *Nucleic Acids Res*, **6**, 3543-3557.

8. FAQ

Question: How to start a design?
Reply: Make you sure you have all requirements; query nucleotide sequence(s) in fasta format and blast-formatted nucleotide database(s). Enter the criteria for oligonucleotide length, T_m and T_m range (you can use the “Best” button to estimate the T_m of your sequence(s)), the blast database and finally the directory in which the results will be saved. Then launch the design process by clicking the “Run” button.
You can also add optional criteria such as the distance from 3’ end or specific positions within the sequence, number of oligonucleotide to retain in the results, GC content limits, prohibited sequences and advanced criteria such as Step, Specificity cutoffs and BLAST options. At the end of the design, you can generate a result file (tab separated), which can be read by almost all spreadsheet programs, containing all best results using the “Save Result” button in the Batch mode. You can also visualize all results using the “Result Panel”.

Q: How to combine multiple designs?
R: Run the design twice for your queries and select for each design a different directory to save the results. Then you can use the X-Selection (available in “Batch mode”) to cross the results of two designs. In “Result Panel”, select the different directories to visualize all the results in the same window.

Q: I have a multi CPU server. Can I launch many CADO4MI on the same set?
R: Yes you can. Simply launch twice (or more) the program and select all your queries (no need to separate your set into non-overlapping set) and run the design. CADO4MI uses a temporary file (“.working”) to check if the same query is being used or not. When one of the running programs will encounter one query that has already been designed (by another run of CADO4MI) it will ask you if you want to redesign it. Simply reply “No” once for each run of CADO4MI.

Q: How to save all my results in one file?
R: The best results for all your queries can be saved in a tab-delimited file with oligonucleotide characteristics (positions, T_m …) by using the function “Save Results” available in the “Batch Mode”.

Q: How to redesign without recalculating the blast?
R: Select your query and enter the new parameters (e.g. another range of T_m), select the same output directory and instead of selecting an existing blast database simply enter the word “none” and it will redesign by keeping the previous blastn file.

Q: I have redesigned oligonucleotide for a query using different criteria. Is it possible to update the first design with some oligonucleotides and then delete the second one?
R: Yes, load the redesign and use “Update another design” button. You will need to give the directory of the design to complete and then to manually select the oligonucleotide you want to add to this directory. Please choose to update the log file if asked. It is recommended to update the results done in the same database and not to update results coming from two distinct databases.
