# Supplementary material for: Design and evaluation of Actichip, a thematic microarray for the study of the actin cytoskeleton
Source: BMC Genomics. 2007 Aug 29;8:294. doi: 10.1186/1471-2164-8-294 (PMC2077341; doi:10.1186/1471-2164-8-294)
Supplement: Additional file 5 — Experimental protocols for transcriptome analysis. [file 1471-2164-8-294-S5.pdf]

Gene expression profiling using two-color microarrays

Actichip microarrays

To generate labelled single-stranded cDNA target, 2 µg of poly(A+) RNA purified from MCF-7 cells or human skeletal-muscle were reverse-transcribed for 2 h at 42°C in the presence of 2 µg oligo(dT) primers (Invitrogen, Merelbeke, Belgium), 1x first-strand synthesis buffer (Invitrogen), 10 mM DTT, dNTP mix (25 mM dATP, 25 mM dCTP, 25 mM dGTP, 15 mM dTTP, 10 mM amino allyl-dUTP), 60 units RNAse OUT (Invitrogen), and 400 units Superscript II reverse transcriptase (Invitrogen). RNA was hydrolysed with 200 mM NaOH and 100 mM EDTA for 15 min at 65°C, then neutralised with 545 mM Tris-HCl pH 7.4. First strand cDNA was purified from unincorporated amino-allyl-dUTPs on QIAquick PCR purification columns (Qiagen, Venlo, the Netherlands) acccording to manufacturer's instructions, except that QIAquick wash buffer was replaced with 5 mM K^+ phosphate buffer pH 8.5 containing 80 % ethanol, and cDNA was eluted with 4 mM K^+ phosphate buffer pH 8.5. Eluted cDNA precipitated for 1h at -80 °C in the presence of 20 µg glycogen, 110 mM sodium acetate pH 5.2, and 75 % ethanol. Precipitated cDNA was recovered by centrifugation, washed with cold 75 % ethanol, resuspended in 8 µl of 0.1 M Na_2CO_3 buffer pH 9.0, and then mixed with either Alexa fluor 555 or Alexa fluor 647 NHS-ester (Invitrogen) previously dissolved in 2 µl of DMSO. The mixture was incubated for 1 h at room temperature in the dark, and labeled cDNA targets were purified on QIAquick PCR purification columns, combined at equimolar concentrations as assessed using a ND-1000 spectrophotometer (Nanodrop, Wilmimgton, DE, USA) and then precipitated as described above. Pellets were washed once with cold 75 % ethanol, and then resuspended in 24 µl hybridisation buffer (25 % formamide, 5x SSC, 0.1 % SDS) containing 10 µg human Cot-1 DNA (Invitrogen) and 10 µg Salmon sperm DNA (Invitrogen). Before use, Actichip microarrays were washed 2 min in 2x SSC, 0.1 % sarcosyl, 2 min in 2x SSC, and then 2 min

in boiling milliQ water. The slides were cooled at room temperature for 10 s in milliQ water, then fixed in ice cold 100 % ethanol for 2 min, and finally dried by centrifugation. The labeled cDNA mix was applied into the hybridisation cell delimited by a 22 x 25 mm Lifterslip (Erie Scientific, Portsmouth, NH, USA). The hybridisation was carried out at 42 °C for 20 h in a Slidebooster 800 (Advalytix, Brunnthal, Germany) with a regular microagitation of the sample at maximum power and a pulse/pause ratio of 3:7 s. Following incubation, the slides were washed at room temperature in 2x SSC, 0.1 % SDS preheated at 42 °C for 10 min, followed by 1x SSC for 10 min and then 0.5x SSC for 10 min, before drying by centrifugation.

Human pangenomic array

Whole human genome microarrays containing 21521 70-mer oligonucleotides (Operon human library version 2.0) spotted onto Corning UltraGAPS slides were manufactured by the genomics laboratory at the university medical center of Utrecht (UMCU, The Netherlands). Just before use, the slides were washed by vigourous shaking for 1 min in 5x SSC, 0.1 % SDS at room temperature. They were incubated for 45 min at 42 °C in 5x SSC, 25 % formamide, 0.1 % SDS, 1 % bovine serum albumin, then dipped five times in room temperature milliQ water followed by isopropanol, before drying by centrifugation. Alexa fluor-labelled cDNA targets were prepared as described above, combined at equimolar concentrations in a final volume of 65 µl hybridisation buffer. Hybridisation was carried out as mentionned above using a 25x60 mm Lifterslip. Following incubation, the slides were washed at room temperature in 1x SSC, 0.2 % SDS for 4 min, then in 0.1x SSC, 0.2 % SDS for 4 min, followed by 0.1x SSC for 4 min, and finally 0.1x SCC for 1 min under vigourous shaking before drying by centrifugation.

Gene expression profiling using single color microarrays

Sample labelling, hybridisation and staining were carried out at the Institut de Génétique et de Biologie Moléculaire et Cellulaire (IGBMC, Strasbourg, France) according to the eukaryotic target preparation protocol in the Affymetrix technical manual for Genechip expression analysis (Affymetrix, Santa Clara, CA, USA). In summary, 200 ng of purified poly(A+) RNA were used in a 20 µl first strand reaction with 200 units Superscript II (Invitrogen) and 100 pmoles (dT)-T7 primer (5'-GGCCAGTGAATTGTAATACGACTCACTATAGGGAGGCGG(T)24-3') in 1x first strand buffer (Invitrogen) with a 42 °C incubation for 1 h. Second strand synthesis was carried out by the addition of 40 units *E. coli* DNA Polymerase (Invitrogen), 2 units *E. coli* RNAse H (Invitrogen), 10 units *E.coli* DNA Ligase (Invitrogen) in 1x second strand buffer (Invitrogen) followed by incubation at 16 °C for 2 h. The mix was supplemented with 10 units *E. coli* T4 DNA Polymerase, and was further incubated for 5 min at 16 °C. Upon addition of 5 µM EDTA, the second strand synthesis reaction was purified using the Genechip sample cleanup module (Affymetrix) according to the manufacturer's protocol. The purified cDNA was amplified using Genechip expression 3'-amplification in vitro transcription kit (Affymetrix) according to the manufacturer's protocol to produce biotin-labelled cRNA (compliment RNA) that was further purified on a Genechip cleanup module. Twenty µg of cRNA were fragmented in 40 µl 1x fragmentation buffer (40mM Tris-acetate pH 8.1, 100 mM KOAc, 30 mM MgOAc) at 94 °C for 35 min. The fragmented labelled cRNA was resuspended in 250 µl 1x hybridisation buffer containing 100 mM MES, 1 M (Na+), 20 mM EDTA, 0.01 % Tween 20, 10 % DMSO, 0.5 mg/ml acetylated BSA, 0.1 mg/ml herring sperm DNA, 50 pM control oligonucleotide B2, and control transcripts bioB 0.75 pM, bioC 2.5 pM, bioD 12.5 pM, and cre 50 pM. 130 µl of the hybridisation cocktail containing 6.5 µg of labeled cRNA were hybridised to human genome arrays HG-U133A 2.0 (Affymetrix) for 16 h at 45°C according

to the manufacturer's protocol. The hybridised arrays were washed and stained with streptavidin-phycoerythrin (Invitrogen), and signal was amplified with biotinylated anti-streptavidin antibodies (Sigma, Bornem, Belgium) using a Genechip fluidics station 400 (Affymetrix) according to the manufacturer's protocol. The arrays were scanned using a Genechip scanner 3000 (Affymetrix) at a resolution of 1.56 µm.
