# Supplementary material for: Design and evaluation of Actichip, a thematic microarray for the study of the actin cytoskeleton
Source: BMC Genomics. 2007 Aug 29;8:294. doi: 10.1186/1471-2164-8-294 (PMC2077341; doi:10.1186/1471-2164-8-294)
Supplement: Additional file 7 — GID and pCover parameters. Sequences of the Actichip targets (GenBank mRNA sequences) were compared to the sequences (GenBank and RefSeq mRNA sequences) provided by Affymetrix ("target sequence") and the genomics laboratory at the university medical center of Utrecht (UMCU, The Netherlands) relative to the Operon probe set. This comparison was performed through sequence alignment using the BLASTN program and calculation of two parameters, a global percent identity (GID) and a percent coverage (pCover). GID corresponds to the percentage of global identity between the query sequence (Qseq; Affymetrix or Operon target sequence) and one sequence (SeqB; Actichip target sequence) in the BLAST output. GID is determined by considering all best scoring alignments (MSP) between QSeq and SeqB and is computed as the ratio of the total number of identical residues (TNAR) to the total number of residues (TNRM) in all these alignments. pCover refers to the percentage of sequence coverage between QSeq and SeqB in the BLAST output. The coverage corresponds to the extended overlapping regions between QSeq and SeqB considering all the regions aligned between the two sequences. pCover is computed as the ratio of the sum of all identical residues (NAR) to the number of residues in coverage (NRC). [file 1471-2164-8-294-S7.pdf]

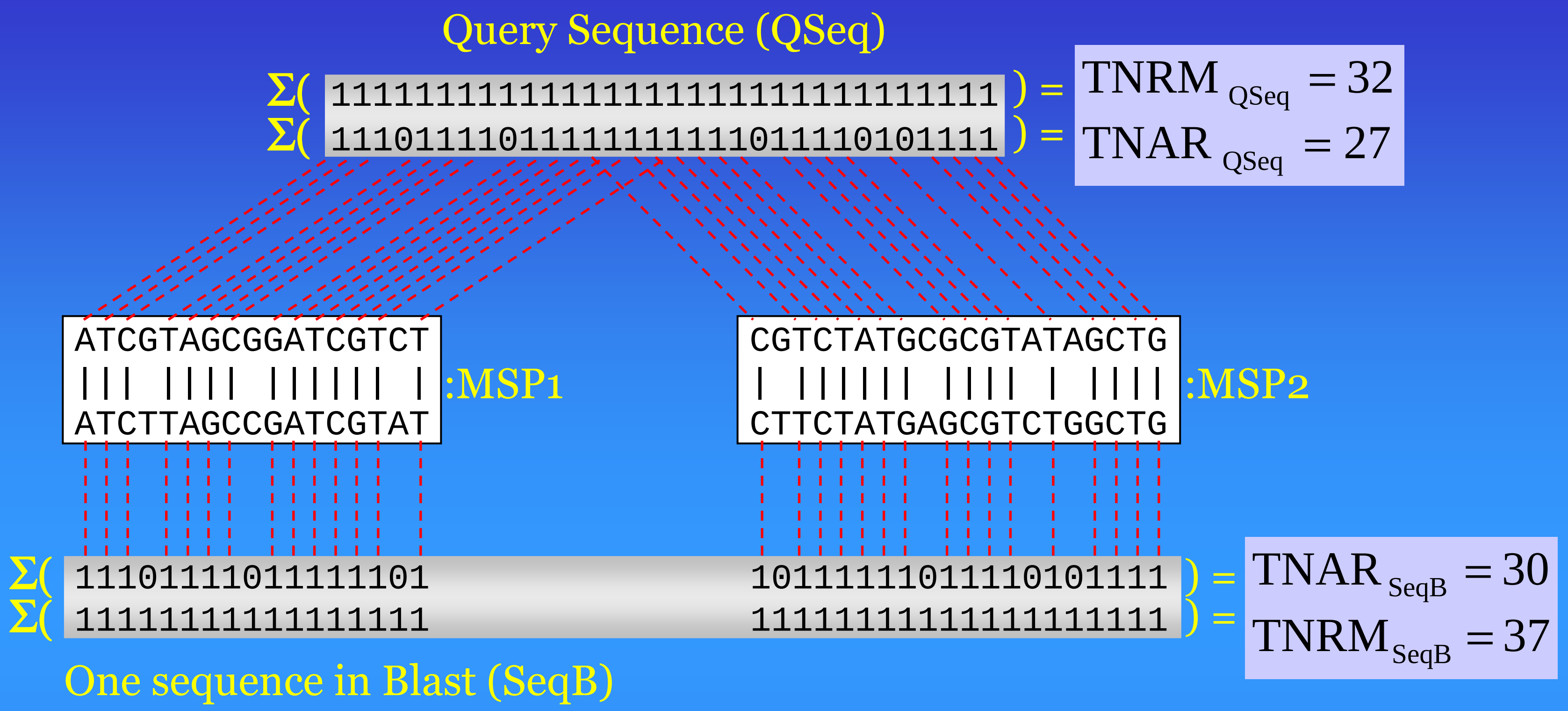

$$GID_{SeqB} = \frac{TNAR_{SeqB}}{TNRM_{SeqB}} \times 100 = \frac{30}{37} \times 100 = 81.1\%$$

TNAR: Total Number of Aligned Residue

TNRM: Total Number of Residue in MSP (*Maximum Segment Pair*)

GID: Global Percent Identity

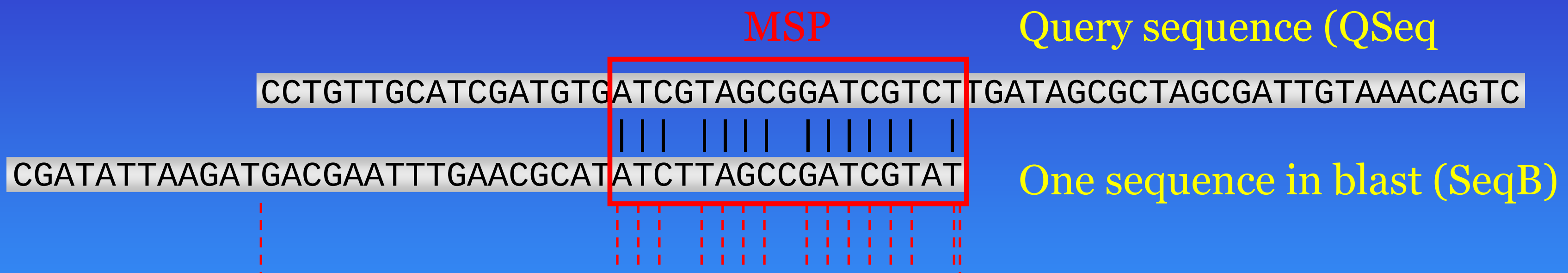

Σ(11101111011111101) = NAR (=14)

Σ(11111111111111111) = NRM (=17)

Σ(1111111111111111111111111111111111) = NRC (=34)

$$pCover = \frac{NAR}{NRC} \times 100 = \frac{14}{34} \times 100 = 41.2\%$$

NAR: Number of aligned residue in MSP (*Maximum Segment Pair*)

NRM: Number of residue in MSP

NRC: Number of residue in coverage

pCover: Percent coverage between the 2 sequences.
